# The current status of nano-hydrogel preparations for osteochondral repair: Systematic Review

**DOI:** 10.3389/fbioe.2025.1611522

**Published:** 2025-07-01

**Authors:** Abebe Feyissa Amhare, Lichun Qiao, Huan Deng, Jinyan Lin, Jun Wang, Wei Wang, Jing Han

**Affiliations:** ^1^ Comprehensive Orthopedic Surgery Department, The Second Affiliated Hospital of Xi’an Jiaotong University, Xi’an, China; ^2^ School of Public Health, Xi’an Jiaotong University Health Science Center, Xi’an, Shaanxi, China; ^3^ Department of Joint Surgery, Honghui Hospital, Xi’an Jiaotong University, Xi’an, Shaanxi, China

**Keywords:** nano-hydrogel, osteochondral repair, tissue engineering, biomaterials, osteochondral

## Abstract

**Background:**

Osteochondral defects, involving both cartilage and subchondral bone, remain clinically challenging due to the poor intrinsic healing capacity of cartilage and the limited durability of traditional treatments. This systematic review aims to evaluate current advancements in nano-hydrogel formulations for osteochondral repair, focusing on their composition, preparation methods, mechanical properties, biocompatibility, and regenerative outcomes.

**Methods:**

Following the Preferred Reporting Items for Systematic Reviews and Meta-Analyses (PRISMA) guidelines, a comprehensive literature search was conducted across PubMed, Web of Science, and Scopus. Eligible studies were screened based on predefined inclusion and exclusion criteria. The methodological quality and risk of bias of included studies were assessed using CAMARADES checklist, which considered factors such as randomization, blinding, animal welfare compliance, outcome reporting, and study reproducibility. Data synthesis was performed through structured tabulation and subgroup stratification by scaffold structure (single-phase, bilayered, trilayered, gradient), formulation type (injectable vs. preformed), and polymer origin (natural, synthetic, hybrid).

**Results:**

A total of 41 studies were included, encompassing both *in vitro* and *in vivo* models, with participant numbers ranging from small animal models (e.g., rabbits, rats) to larger preclinical systems. Studies varied in scaffold design, bioactive integration, and fabrication techniques. Most nano-hydrogels demonstrated high biocompatibility, tunable degradation, and enhanced tissue integration. However, heterogeneity in design parameters, lack of standardized outcome measures, and variable reporting quality limited direct comparisons.

**Conclusion:**

Nano-hydrogels show strong potential as biomimetic scaffolds for osteochondral repair, offering customizable mechanical and biological properties. Nevertheless, the evidence base is limited by study heterogeneity, moderate risk of bias, and lack of standardized protocols, which complicates direct comparison and clinical extrapolation. Future work should focus on long-term validation, functional outcome measures, and development of smart, adaptive materials to support clinical translation.

## 1 Introduction

Osteochondral defects, characterized by damage to both cartilage and the underlying bone, present a significant clinical challenge due to the limited regenerative capacity of cartilage tissue and the complex architecture of the osteochondral unit (Mano and Reis, 2007; Dinoro et al., 2019; Davis et al., 2021; Liu et al., 2021a). These defects are commonly caused by trauma, osteoarthritis, and other degenerative conditions, leading to pain, reduced mobility, and a decreased quality of life (Verhagen et al., 2003; Martin et al., 2007; Liu et al., 2020). Traditional treatments, such as microfracture surgery, autologous chondrocyte implantation, and osteochondral allografts, often fail to provide long-term solutions, particularly for larger lesions, due to complications such as donor site morbidity, limited graft availability, and incomplete integration with host tissues (Hjelle et al., 2002; Cavendish et al., 2019; Chahla et al., 2019). Consequently, there is a critical need for innovative therapeutic strategies that can effectively promote the regeneration of both cartilage and subchondral bone in a coordinated manner (De Leon-Oliva et al., 2023; Li et al., 2023b).

Recent advances in tissue engineering and regenerative medicine have highlighted the potential of biomaterials to overcome the limitations of conventional therapies (Lynch et al., 2021; Zhang et al., 2021; Cao and Ding, 2022; Luo et al., 2022). Among the various biomaterials explored, nano-hydrogel systems have garnered significant attention due to their unique physicochemical properties and versatility (Chander et al., 2021; Ahmad et al., 2022; Sethi et al., 2023; Rana and De la Hoz Siegler, 2024). Nano-hydrogels are three-dimensional, water-swollen polymeric networks that can be engineered to mimic the native extracellular matrix (ECM) of osteochondral tissues (Liu and Hsu, 2018; Zengin et al., 2021; Hwang and Lee, 2024). Their nano-scale features, high surface area, and tunable mechanical properties make them ideal candidates for supporting cell adhesion, proliferation, and differentiation (Quazi and Park, 2022; Hwang and Lee, 2024). Additionally, nano-hydrogels can be easily functionalized to deliver therapeutic agents, such as growth factors, cytokines, and nanoparticles, in a controlled and sustained manner, further enhancing their regenerative potential (Lee, 2018; Soni et al., 2022).

The design and development of nano-hydrogels for osteochondral repair involve several key considerations, including mechanical strength, biodegradability, biocompatibility, and the ability to support dual regeneration of cartilage and bone (Yue et al., 2020; Xiang et al., 2022; Yao et al., 2023). Successful regeneration requires a scaffold that not only mimics the structural and functional properties of the native tissue but also degrades at a rate that matches the pace of tissue formation, thereby providing support throughout the healing process (Yue et al., 2020; Hwang and Lee, 2024). Furthermore, the incorporation of bioactive molecules that can modulate the local cellular environment is essential for promoting chondrogenic and osteogenic differentiation, ensuring effective integration of the scaffold with host tissues (Yue et al., 2020; Xiang et al., 2022).

While numerous studies have reported the development of nano-hydrogel systems for osteochondral repair, there remains a lack of comprehensive understanding regarding the optimal design parameters and functionalization strategies (Wang et al., 2022b). Additionally, the variability in experimental models and evaluation criteria across studies has made it challenging to compare outcomes and draw definitive conclusions about the efficacy of different approaches (Hwang and Lee, 2024). To address these gaps, this systematic review aims to provide a detailed overview of the current status of nano-hydrogel preparations for osteochondral repair, with a focus on their composition, preparation methods, mechanical properties, biocompatibility, and *in vitro* and *in vivo* efficacy.

This review analyzes and synthesizes findings from recent literature, highlighting key advancements and identifying existing challenges in the field. It offers insights into the design principles that guided the development of next-generation nano-hydrogel systems, ultimately contributing to the advancement of more effective and reliable therapeutic solutions for osteochondral defects.

## 2 Materials and methods

This systematic review was performed according to the Preferred Reporting Items for Systematic Reviews and Meta-Analyses (PRISMA) guidelines (Moher et al., 2009; Moher et al., 2015). A protocol was specified and registered on the database International Prospective Register of Systematic Reviews (PROSPERO) (registration number CRD42024586563) and is available from: https://www.crd.york.ac.uk/prospero/#myprospero.

### 2.1 Search strategy

A comprehensive search was conducted across three English-language databases: PubMed, Scopus, and Web of Science. The search focused on identifying studies related to nano-hydrogel systems for osteochondral repair. Search terms included combinations of MeSH and free-text keywords: (“nanohydrogel” OR “nanogel” OR “nano-hydrogel scaffold” OR “nanoscale hydrogel” OR “nano-sized hydrogel” OR “nanocomposite hydrogel”) AND (“osteochondral repair” OR “cartilage regeneration” OR “cartilage repair” OR “osteochondral defect”). Filters were applied to include only English-language publications. A detailed list of search terms and strategies for each database is provided in Supplementary Table S1.

**FIGURE 1:**
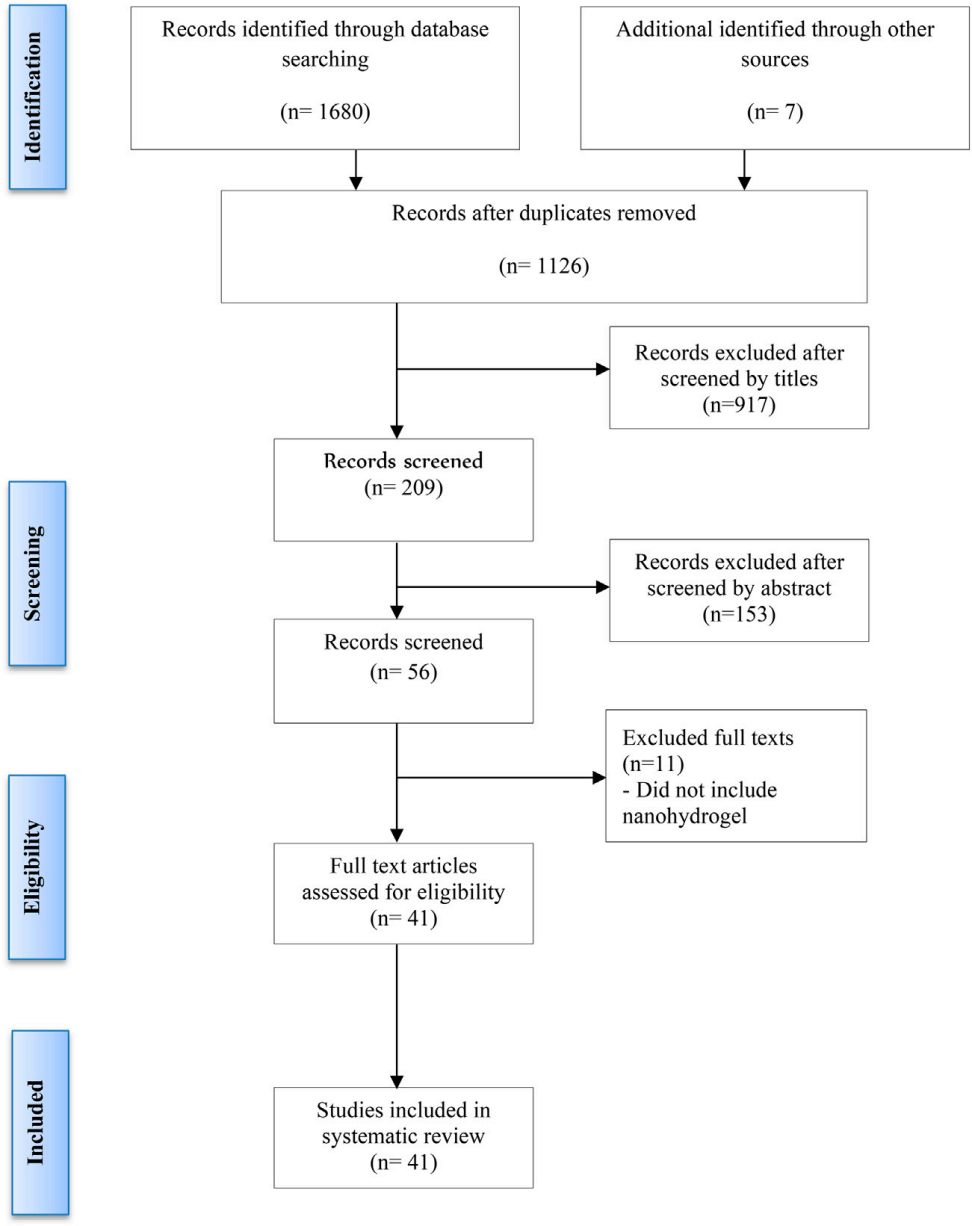
Flow diagram of the study selection process.

Additionally, reference lists of retrieved articles were manually reviewed to identify any further relevant studies. Two authors (AFA and LQ) independently screened titles and abstracts to assess eligibility based on the inclusion criteria. Full-text articles were further reviewed to exclude any duplicates or studies that did not meet the criteria (Figure 1). Discrepancies were resolved through discussion with a third reviewer (JH). The last update search was conducted on 29 September 2024.

### 2.2 Focused question

This systematic review was performed to address the following focused question: “What is the current status of nano-hydrogel preparations in promoting osteochondral repair, specifically regarding their composition, preparation methods, mechanical properties, biocompatibility, and therapeutic efficacy?”

### 2.3 Selection criteria

To ensure the inclusion of high-quality and relevant studies, specific eligibility criteria were established prior to the screening process. Studies were included if they were original research articles published in peer-reviewed journals, written in English, and focused on the preparation and application of nano-hydrogel systems specifically for osteochondral or cartilage repair. Eligible studies were required to provide sufficient detail on the hydrogel’s composition, crosslinking or functionalization strategies, and report at least one form of biological or functional evaluation, whether *in vitro*, *ex vivo*, or *in vivo*.

Studies were excluded if they were review articles, conference abstracts, dissertations, clinical case reports, editorials, or other forms of grey literature. Additionally, publications that did not focus on osteochondral repair, or those that lacked essential data on hydrogel characterization or biological performance, were omitted. There were no restrictions on publication year; however, only articles published in English were considered. These criteria were designed to ensure methodological rigor and relevance to the focused research question.

### 2.4 Screening methods and data extraction

Titles and abstracts were screened by two independent reviewers (AFA and LQ), followed by full-text assessments for studies that met the inclusion criteria. Disagreements on study eligibility were resolved through consultation with a third reviewer (JH). The extracting data were following PICO (P: sources, I: interventions, C: control study, O: outcomes) standards.

The data extraction process focused on gathering information about general study characteristics, including nano-hydrogel composition, types of nanoparticles, preparation methods, crosslinking strategies, and controlled release mechanisms. It also covered mechanical and bioactivity properties, such as mechanical strength, degradation rates, biocompatibility, swelling ratios, and functionalization aspects. For *in vitro* studies, details on cell types, culture conditions, cell viability, and proliferation were collected. *In vivo* studies were evaluated based on animal models, group allocation, implantation techniques, histological assessments, and outcomes related to subchondral bone and cartilage regeneration, including immunohistochemical findings, inflammation, infection, and hydrogel degradation. Lastly, the extraction included identification of research limitations and recommendations for future studies, ensuring a comprehensive overview of each study’s approach and findings.

### 2.5 Quality assessment and analysis of the data

The methodological quality of the included studies was evaluated using a customized CAMARADES checklist, which I adapted to better assess the relevance of each study (Macleod et al., 2004). The adapted checklist incorporated 11 key criteria to assess study relevance: (1) publication in a peer-reviewed journal, (2) random allocation to treatment or control groups, (3) blinded outcome assessment, (4) Control of the temperature in the animal facilities, (5) use of appropriate controls, (6) adequate sample size, (7) clear description of the animal model, (8) adherence to animal welfare guidelines, (9) reproducibility and replication of findings, (10) thorough outcome reporting, and (11) disclosure of any potential conflicts of interest. Given the nature of the data, analysis was conducted descriptively, as the variability across studies precluded meta-analysis.

## 3 Results and discussion

### 3.1 Search outcomes

**FIGURE 2:**
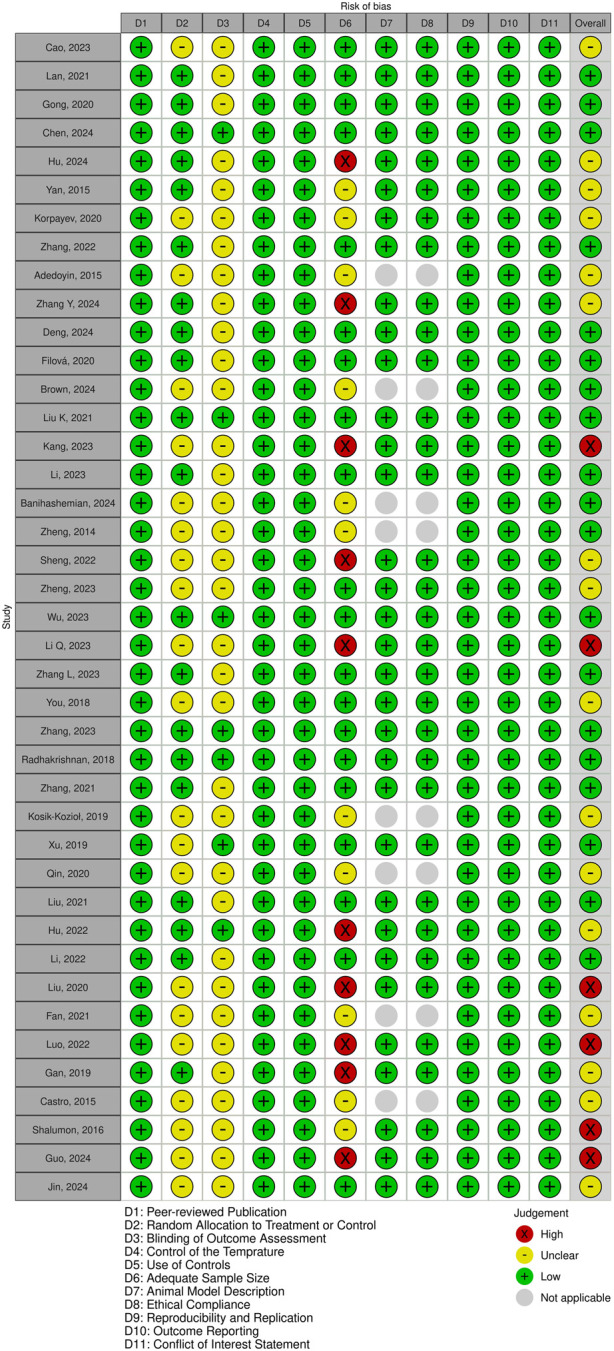
Quality assessment of included studies using a modified CAMARADES checklist.

**FIGURE 3 F3:**
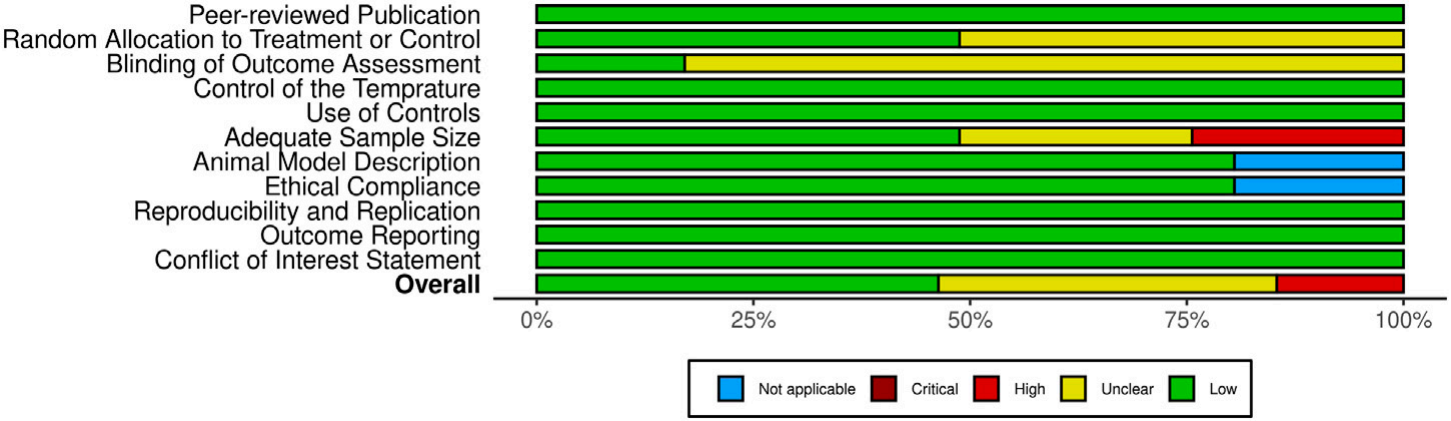
Overview of risk of bias assessment for included studies using a modified CAMARADES checklist.

Following the removal of duplicates, a total of 1,126 unique publications were identified through database screening. Title and abstract screening narrowed these to 56 articles for full-text evaluation. After applying the inclusion criteria, 11 studies were excluded. Consequently, 41 studies were included in this systematic review (Figure 1). Of these, 34 studies employed both *in vitro* and *in vivo* methodologies, while seven were limited to *in vitro* experiments (Adedoyin et al., 2015; Castro et al., 2015; Kosik-Kozioł et al., 2019; Qin et al., 2020; Fan et al., 2021; Banihashemian et al., 2024; Brown et al., 2024). The assessment of bias showed a spectrum from low to high risk, and detailed findings on methodological quality are illustrated in Figures 2,3, [Fig F3].

### 3.2 Nano-hydrogel composition and preparation methods

The studies summarized in Table 1 highlight the structural and compositional diversity of nano-hydrogel systems used for osteochondral repair. These range from simple, single-phase injectable formulations to more complex preformed multilayered scaffolds—each engineered to address distinct mechanical and biological requirements. Scaffold configurations were stratified into single-phase, bilayered, trilayered, and gradient systems. Many bilayered and trilayered constructs were designed to emulate the zonal architecture of osteochondral tissue, allowing site-specific modulation of chondrogenesis and osteogenesis.

**TABLE 1:** General study information and methods.

Nano-hydrogel composition	Nanoparticles used	Formulation type	Polymer origin	Preparation methods and crosslinking strategies	Controlled release	References
Single-phase hydrogel: CuTA@SF hydrogel	Cu nanoparticles	Injectable	Natural	CuTA synthesized by combining Cu nanoparticles with TA; incorporated into SF hydrogel; enzymatically crosslinked using HRP and H_2_O_2_	TA release from CuTA@SF hydrogel monitored using BCA assay	Cao et al. (2023)
Bi-layer scaffold: PVA/Col-II/CS (upper), PVA/BCP/CNTs (lower)	BCP, CNTs	Preformed	Hybrid	Freeze–thawing method used to fabricate bi-layer hydrogels, with physical crosslinking	Not explicitly mentioned	Lan et al. (2021)
Bi-layer scaffold: IL-4-loaded GelMA (upper), PCL-HA (lower)	HA	Preformed	Hybrid	The bi-layer scaffold was fabricated using two 3D printing techniques: DLP for GelMA and FDM for PCL-HA; physical crosslinking for PCL-HA	IL-4 release from GelMA scaffold monitored over 168 h	Gong et al. (2020)
Trilayered scaffold: GL-HPKGN (upper), GL-GMA (middle), GL-HP/GMAAT (lower)	HA	Preformed	Natural	Enzyme crosslinking for upper layer (KGN-Gelatin), photo-crosslinking for middle layer (GMA-Gelatin), dual-crosslinking for lower layer (Atorvastatin-Gelatin)	KGN and AT grafted into the hydrogels, providing sustained release	Chen et al. (2024)
LiMn_2_O_4_ nanozyme-functionalized bilayer hydrogel scaffold	LiMn_2_O_4_ nanozyme, nHA	Preformed	Hybrid	Cartilage layer crosslinked via UV light; subchondral layer crosslinked by Zn^2+^ and UV light	LiMn_2_O_4_ nanozyme was gradually released, reaching 73.2% release by Day 30	Hu et al. (2024)
Bilayered scaffold: top silk fibroin layer; bottom silk-nano (CaP) layer	NanoCaP	Preformed	Natural	Silk-nanoCaP layer prepared with 16 wt% SF and CaP particles; the scaffold was created by salt-leaching and freeze-drying techniques	Not explicitly mentioned	Yan et al. (2015)
Tri-layer scaffold: Chi/Col I + II/nHA	nHA	Preformed	Natural	Freeze-drying for bone layer; thermal gelation for calcified cartilage and cartilage layers	Not explicitly mentioned	Korpayev et al. (2020)
Bi-layer scaffold: mPEG-b-PLV thermogel	HA	Preformed	Hybrid	mPEG-b-PLV thermogel was prepared via ring-opening polymerization; PLGA/HA scaffold was prepared via salt-leaching with HA particles	Sustained release of KGN from thermogel and BMP-2 from PLGA/HA scaffold	Zhang et al. (2022b)
Single-phase hydrogel: p (NiPAAm-co-GMA)/PAMAM	Fe_3_O_4_	Injectable	Synthetic	Mixed p (NiPAAm-co-GMA) and PAMAM; dual gelation achieved via thermal and chemical crosslinking	Not explicitly mentioned	Adedoyin et al. (2015)
Hybrid scaffold: Zn-AlgMA hydrogel coating DCPD-coated porous Mg alloy	Zn^2+^ in the Zn-AlgMA	Preformed	Hybrid	Zn-AlgMA hydrogel prepared using zinc ion crosslinking and UV light crosslinking	Controlled release of Mg^2+^ and Zn^2+^ from Zn-AlgMA	Zhang et al. (2024)
Bi-layer scaffold: DE-incorporated GelMA	DE microparticles (Si ions)	Preformed	Hybrid	GelMA and DE-incorporated scaffolds fabricated using 3D printing technology; DE microparticles filtered and incorporated into GelMA solution	Continuous release of Si ions from DE microparticles	Deng et al. (2024)
Composite gel containing PCL-chit-PEGb-antiCD44 microparticles	PCL-CS microparticles	Injectable	Hybrid	PCL-CS nanofibers prepared by electrospinning, then cryogenically grinded into microparticles, followed by modification with PEG and anti-CD44 antibody	Not explicitly mentioned	Filová et al. (2020)
Single- and dual-layer hydrogel–PCL composite scaffold: Heparin-containing PEGDA hydrogel	Heparin (sulfated glycosaminoglycan)	Preformed	Hybrid	Hydrogel synthesized with PEGDA, dithiothreitol for hydrolytic degradation; scaffolds printed using selective laser sintering	Sustained release of heparin-bound small molecules over 14 days	Brown et al. (2024)
Bi-layer scaffold: Upper (HLC-HA), Lower (HLC-HA-HAP)	nHA	Preformed	Natural	Liquid phase synthesis, freeze-drying, and chemical crosslinking with EDC/NHS	Not explicitly mentioned	Liu et al. (2021a)
Bi-layer-like: GTU-Fe hydrogel film with spatial *in situ* deposition of KGN@PDA (top) and miRNA@CaP (bottom)	KGN@PDA and miRNA@CaP	Preformed	Natural	*In situ* deposition of drug and gene nanoparticles on the supramolecular-assembled UPy-GelMA hydrogel	Controlled release of KGN and miR-26a; cumulative release over 7 days	Kang et al. (2024)
Bi-layer scaffold: ECM hydrogel-coated ECM/PCL (upper cartilage) + MgO@PDA/PCL (lower bone)	MgO nanoparticles	Preformed	Hybrid	3D-printed PCL scaffold incorporating MgO@PDA for the subchondral bone layer and ECM hydrogel for the cartilage layer	Sustained release of Mg^2+^ from the MgO@PDA	Li et al. (2023a)
Bi-layer scaffold: Alginate-nHA with CS-hyaluronic acid	nHA	Preformed	Natural	Alginate and nHA scaffold for subchondral phase; CS-HA scaffold for chondral phase; both layers assembled using fibrin glue	Not explicitly mentioned	Banihashemian et al. (2024)
Triple-phase hydrogel: *In situ* synthesized nHA/collagen/alginate hydrogel	nHA	Injectable	Natural	*In situ* synthesis of nHAp in collagen gel followed by addition of alginate and crosslinking with Ca^2+^ ions	Not explicitly mentioned	Zheng et al. (2014)
Single-phase: Nanosilicate-reinforced silk fibroin (SF-MMT) hydrogel	Montmorillonite (MMT)	Injectable	Natural	Enzymatically crosslinked SF-MMT hydrogel prepared by mixing SF with MMT and crosslinking via HRP and H_2_O_2_	Not explicitly mentioned	Sheng et al. (2022)
Single-phase: High-porosity GelMA hydrogel with 5% methacrylated n-HApMA and ADSCs	nHA and nHAMA	Injectable	Natural	Surface modification of nHA using alkylation; bio-inks prepared by incorporating nHAMA and adipose-derived stem cells (ADSCs) into high-porosity GelMA	Not explicitly mentioned	Zheng et al. (2023)
Single-phase: GelMA hydrogel loaded with IGF-1 bioactive supramolecular nanofibers (BSN-GelMA)	IGF-1 bioactive supramolecular nanofibers (IGF-1bsn)	Injectable	Hybrid	Supramolecular nanofibers synthesized via solid-phase peptide synthesis; incorporated into GelMA hydrogel using photo-initiator LAP	Sustained release of IGF-1bsn from hydrogel for enhanced regeneration	Wu et al. (2023)
Bi-layer scaffold: Double-network hydrogel scaffold	hADSC-derived exosomes	Preformed	Hybrid	3D printing with dECM bioinks (Hydrogel-DCM and Hydrogel-DBM) incorporating exosomes; crosslinked with GelMA and HA derivatives	Sustained release of exosomes from the hydrogel scaffold over 24 days	Li et al. (2023b)
Multileveled hierarchical hydrogel with continuous nHA gradients	Superparamagnetic HA (MagHA) nanorods	Preformed	Hybrid	Hydrogel matrix fabricated using 3D printing; MagHA gradient formed under magnetic force; acrylated disodium pamidronate (ADP) used for covalent bonding with GelMA hydrogel	Not explicitly mentioned	Zhang et al. (2023a)
Bilayered hydrogel composed of nHA, CS, and PEGDA	nHA	Preformed	Natural	Hydrogels prepared via Schiff-base reaction (CEC + OHA) and PEGDA photocrosslinking for osteochondral scaffold construction	Not explicitly mentioned	You et al. (2018)
Bi-layer scaffold: KGN-loaded GelMA hydrogel	HA	Preformed	Natural	GelMA hydrogels were crosslinked with LAP under UV light; PCL scaffold was 3D printed and coated with HA using alternate soaking technology	Sustained release of KGN from GelMA hydrogels	Zhang et al. (2023b)
Gradient scaffold: Alginate/PVA SIPN hydrogel formed *in situ*	nHA and chondroitin sulfate	Injectable	Hybrid	*In situ* semi-interpenetrating network (SIPN) hydrogel with gradient CS and nHA integration via wet chemical precipitation and calcium crosslinking	Not explicitly mentioned	Radhakrishnan et al. (2018)
Gradient scaffold: 3D printed gradient nHA hydrogel scaffold	nHA	Preformed	Hybrid	3D bioprinting of SA/AM (sodium alginate and acrylamide) hydrogels with CaCl_2_ crosslinking and gradient nHA loading via electronic spray method	Not explicitly mentioned	Zhang et al. (2021)
Single-phase: Alginate-GelMA hydrogel with 0.5% β-TCP for modeling calcified cartilage	β-Tricalcium phosphate (TCP)	Preformed	Hybrid	Bioink formulation with 6% GelMA, 4% alginate, and 0.5% TCP microparticles; bioprinted using extrusion-based printing with coaxial needle	Not explicitly mentioned	Kosik-Kozioł et al. (2019)
Single-phase: HGM supramolecular gelatin hydrogel loaded with KGN and/or TGF-β1	Not explicitly used	Injectable	Natural	Hydrogels synthesized using a host-guest macromer approach, with β-cyclodextrin (Ac-β-CD) and GelMA	Sustained release of TGF-β1 and KGN for up to 28 days	Xu et al. (2019)
Bi-layer scaffold: Cartilage layer (PLGA/CS hydrogel with tubular pores), Bone layer (nHA-g-PLGA/CS porous scaffold)	Grafted nano-hydroxyapatite (nHA-g-PLGA)	Preformed	Hybrid	PLGA/CS hydrogel for cartilage layer and nHA-g-PLGA/CS scaffold for subchondral bone prepared using electrostatic interaction and crosslinking via EDC/NHS	Not explicitly mentioned	Qin et al. (2020)
Bi-layer scaffold: GC hydrogel (CK2.1/β-GP/CS) for cartilage and LL37@LDH/CS for bone	Layered double hydroxide (LDH)	Preformed	Hybrid	CK2.1 was incorporated into the GC hydrogel; LL37 was loaded into the LDH/CS scaffold using freeze-drying and chemical modification techniques	Sustained release of CK2.1 from the GC hydrogel	Liu et al. (2021b)
Tri-layer scaffold: CS/Gel/nHA	nHA	Preformed	Hybrid	Multilayer scaffold prepared via iterative layering with crosslinking using NHS/EDC	Not explicitly mentioned	Hu et al. (2022)
Tri-layer gradient scaffold: Gradient nHA hydrogel scaffold	nHA	Preformed	Natural	Fabrication of nHA/GelMA scaffold through 3D printing; multi-layer structure created using sedimentation of nHA and photocrosslinking	Not explicitly mentioned	Li et al. (2022)
Biphasic hydrogel composed of BRH and CRH	β-Cyclodextrin nanoboxes	Injectable	Natural	CRH (HAMA-based) and BRH (GelMA-based) hydrogels prepared via photocrosslinking, with drug nanoboxes for phase-specific delivery	Sustained release of KGN in the CRH and MLT in the BRH	Liu et al. (2020)
Gradient mineralized double-network (DN) hydrogel	HA	Preformed	Natural	Hydrogels prepared using a double-network method, with gradient mineralization achieved through a segmented soaking process	Not explicitly mentioned	Fan et al. (2021)
Bi-layer scaffold: Composed of γ-PGA, CMCS, and BC	nHA	Preformed	Hybrid	Hydrogel prepared using γ-PGA, CMCS, and BC via chemical and physical crosslinking; bioactive ions (Mg^2+^ and Cu^2+^) introduced to cartilage and bone layers	Sustained release of Mg^2+^ and Cu^2+^ for dual regulatory functions	Luo et al. (2022)
Bi-layer scaffold: Mussel-inspired tough hydrogel with *in situ* nHA mineralization	HA	Preformed	Natural	Bilayer hydrogel prepared using a one-pot method; PDA facilitates *in situ* HA mineralization for subchondral bone repair	Sustained release of BMP-2 and TGF-β3 from hydrogel layers	Gan et al. (2019)
Bi-layer scaffold: PEG-DA hydrogel matrix and nHA	nHA	Preformed	Synthetic	3D printing using fused deposition modeling (FDM) to create a biphasic scaffold with nHA in the osseous layer and TGF-β1 in the cartilage layer	Sustained release of TGF-β1 in the cartilage layer over 21 days	Castro et al. (2015)
Bi-layer scaffold: PLGA and nHA	nHA	Preformed	Synthetic	PLGA and PLGA/nHA microspheres were prepared using the oil-in-water emulsion/solvent evaporation method	Not explicitly mentioned	Shalumon et al. (2016)
Tri-layer scaffold: Injectable and self-healing hydrogel (Ta@gel)	TA and HA	Preformed	Hybrid	Injectable and Ta@gel, combined with 3D-printed HA scaffold; BMSCs encapsulated within GelMA microspheres were loaded into Ta@gel	O_2_ consumption by TA maintains a hypoxic microenvironment for 20 days	Guo et al. (2024)
Single-phase hydrogel: GelMA/Eu-HA nanocomposite hydrogel	Eu-HA nanorods	Injectable	Natural	Hydrothermal synthesis of Eu-HA nanorods, incorporated into GelMA hydrogel via UV crosslinking	Gradual release of Eu ions from Eu-HA nanorods	Jin et al. (2024)

BCP, biphasic calcium phosphate; CS, chitosan; DLP, digital light processing; EU-HA, Europium-doped Hydroxyapatite; GelMA, gelatin methacrylate; HRP, horseradish peroxidase; H_2_O_2_, hydrogen peroxide; KGN, kartogenin; nHA, Nano-hydroxyapatite; PCL, polycaprolactone; PDA, polydopamine; PEG-DA, polyethylene glycol diacrylate; PLGA, Poly Lactic-co-Glycolic Acid; hADSC, Human Adipose-derived Stem Cells.

Integration of nanoparticles such as hydroxyapatite (HA), chitosan montmorillonite, silica, and polydopamine (PDA) has been shown to enhance the mechanical integrity, osteoconductivity, and cellular interactions of hydrogels (Shalumon et al., 2016; Gong et al., 2020; Korpayev et al., 2020; Sheng et al., 2022; Hu et al., 2024; Jin et al., 2024). For instance, a study by Cao et al. (2023) utilized Cu-based nanoparticles embedded in a silk fibroin (SF) matrix via enzymatic crosslinking to create a single-phase injectable hydrogel with antioxidative and immunomodulatory properties Similarly, preformed bilayer hydrogels composed of polyvinyl alcohol (PVA), biphasic calcium phosphate (BCP), and carbon nanotubes (CNTs) were fabricated through a freeze-thawing process to generate a gradient interface, mimicking native cartilage–bone transition zones (Lan et al., 2021). These examples illustrate how both formulation type and nanoparticle selection directly influence the functional performance of nano-hydrogels.

The choice of crosslinking strategy is another determinant of scaffold performance, affecting mechanical stability, degradation behavior, and cellular response. Studies included a wide array of crosslinking approaches, enzymatic, photo-initiated, thermal, chemical, ionic, and dual-crosslinking methods, each tailored to the specific polymer systems and application needs (Adedoyin et al., 2015; Xu et al., 2019; Zhang et al., 2022b; Cao et al., 2023; Wu et al., 2023; Chen et al., 2024). For instance, photo-crosslinking has been employed to allow spatially controlled gelation, ideal for constructing gradient or multi-layered hydrogels (Zhang et al., 2024). However as highlighted in multiple reports, optimization is needed to reduce cytotoxicity from residual initiators, which may impact cell viability and tissue integration (Berry et al., 2019; Hu et al., 2019; Tomal and Ortyl, 2020). In terms of polymer origin, systems were broadly classified as natural, synthetic, or hybrid. Natural polymers like chitosan, gelatin (GelMA), alginate, and hyaluronic acid offer favorable biocompatibility and degradation profiles. Synthetic polymers such as PEGDA, PVA, and PLGA provide enhanced mechanical tunability and process control. Hybrid systems, which combine the strengths of both natural and synthetic components, emerged as especially promising in balancing bioactivity with structural integrity, several trilayered and bilayered scaffolds utilized such combinations to achieve distinct zone-specific functions.

Moreover, the application of advanced fabrication methods such as 3D printing, electrospinning, microsphere sintering, and solvent casting enabled precise spatial organization of materials. These techniques facilitated the development of functionally graded scaffolds, often incorporating nano-hydroxyapatite (nHA) or exosome-loaded layers, to mimic the mechanical and biochemical gradients of native osteochondral tissue (Zhang et al., 2022b; Brown et al., 2024). Several preformed multilayered systems were constructed with dual or triple layers, each designed with distinct pore architectures, ion release kinetics, and biofunctional molecules to modulate regeneration in a zone-specific manner.

Collectively, the reviewed studies demonstrate how scaffold architecture (e.g., single-phase, bilayered, trilayered), formulation type (injectable vs. preformed), polymer composition (natural, synthetic, hybrid), nanoparticle inclusion, crosslinking strategy, and fabrication technique can be tailored in concert to engineer next-generation nano-hydrogels for osteochondral repair. This multi-dimensional classification, as summarized in Table 1, provides a comparative framework to inform rational scaffold design and translational scaffold development.

### 3.3 Mechanical properties and degradation behaviour

Mechanical properties are essential for nano-hydrogel systems, particularly for osteochondral repair, where the scaffold must withstand the mechanical stresses of both cartilage and subchondral bone environments. As observed in Table 2, studies report varied mechanical strengths, with compressive moduli ranging from 0.4 MPa (Mpa) to over 73 MPa depending on the hydrogel composition (Gong et al., 2020; Zhang et al., 2022b; Brown et al., 2024; Hu et al., 2024; Kang et al., 2024). For instance, polycaprolactone-hydroxyapatite (PCL-HA) scaffolds have demonstrated compressive moduli as high as 73 ± 1 MPa, while IL-4-loaded GelMA-PCL-HA composites exhibit lower values around 4.7 ± 0.6 MPa (Gong et al., 2020). These scaffold values are within the range of trabecular (cancellous) bone, which exhibits compressive moduli typically between 10 and 200 MPa, depending on site and density. In contrast, the modulus of natural cortical bone is substantially higher, with a longitudinal elastic modulus ranging from 17.2 to 23.2 GPa and a transverse modulus ranging from 10.8 to 13.9 GPa, as demonstrated through multiscale modeling validated by nanoindentation and ultrasound measurements (Hamed et al., 2010). These comparisons highlight the potential of HA-containing scaffolds to approximate native bone behavior in osteochondral repair applications, particularly when enhanced with structural reinforcements like hydroxyapatite.

**TABLE 2:** Mechanical properties and physical characteristics.

Mechanical properties	Degradation rate	Degradation condition (Temp/Env’t)	Swelling ratio	References
Stable mechanical properties; storage modulus (G′) > loss modulus (G″); viscosity increased with TA and CuTA	87.9% remained after 70 days in PBS	In an incubator	Swelling equilibrium reached after 72 h	Cao et al. (2023)
Tensile modulus: 7.14 ± 3 MPa; compression modulus: lower layer (0.081 MPa) > upper layer (0.011 MPa)	Slower degradation; upper layer degraded faster	In an incubator	Upper layer: 586% ± 52%; Lower layer: 151% ± 7.1%	Lan et al. (2021)
Compressive modulus: PCL-HA scaffold: 73 ± 1 MPa; IL-4-loaded GelMA-PCL-HA: 4.7 ± 0.6 MPa	GelMA hydrogels degraded with 23% mass retention by day 56	Body temperature	Not reported	Gong et al. (2020)
Shear modulus: Upper layer (54.4 ± 1.2 Pa), Middle layer (700 ± Pa), Lower layer (1,500 ± Pa)	Upper layer degraded faster; both biodegradable in collagenase	In an incubator	Upper layer: 155.3% ± 12.1%; Lower layer: 123.6% ± 11.9%	Chen et al. (2024)
Compressive modulus of GH@LM + GA@HLM hydrogel was 73.53 kPa	Nearly complete degradation by day 30	In an incubator	Swelling equilibrium reached after 12 h	Hu et al. (2024)
Compressive modulus (wet state): 0.4 MPa; storage modulus up to 0.8 MPa	27% degradation after 7 days in protease XIV solution	In an incubator	Not explicitly mentioned	Yan et al. (2015)
Compressive modulus: Bone layer (42.95 ± 4.3 kPa), calcified cartilage (5.41 ± 0.6 kPa), cartilage (1.49 ± 0.3 kPa)	Not explicitly mentioned	In an incubator	Not explicitly mentioned	Korpayev et al. (2020)
Compressive modulus of PLGA/HA scaffold: 73.53 kPa; pore size increased during degradation	mPEG-b-PLV thermogel showed 48.4% degradation after 30 days	In an incubator	Not explicitly mentioned	Zhang et al. (2022b)
Young’s modulus via unconfined compression; suitable for tissue regeneration	Not explicitly mentioned	In an incubator	Not explicitly mentioned	Adedoyin et al. (2015)
Elastic modulus of Mg scaffold: 0.9–8.8 MPa; Zn-AlgMA improved mechanical stability	Gradual degradation in Hank’s solution	In an incubator	Not explicitly mentioned	Zhang et al. (2024)
Elastic modulus increased from 493.3 Pa (GelMA) to 1,010.2 Pa (20% DE); Young’s modulus increased from 64.2 kPa to 122.7 kPa	Slower degradation with higher DE concentration	In an incubator	Not explicitly mentioned	Deng et al. (2024)
Higher storage modulus with microparticles than fibrin; loss modulus higher in fibrin	Not explicitly mentioned	In an incubator	Not explicitly mentioned	Filová et al. (2020)
Compressive strength varies with porosity: 70% (494 kPa), 80% (100 kPa), 90% (20 kPa)	Degraded within 4 weeks at 20 mol% DTT concentration	In an incubator	Increased fold swelling with higher DTT content	Brown et al. (2024)
Compressive strength: Bilayer (212.11 ± 13.49 kPa) vs. single layer (87.47 ± 13.29 kPa)	Not explicitly mentioned	In an incubator	Bilayer scaffold: 498.74%; Single-layer: 789.08%	Liu et al. (2021a)
Compressive strength of GTU-Fe hydrogel: 2.59 MPa; excellent viscoelasticity	Gradual degradation; sustained release of KGN and miR-26a	In an incubator	Not explicitly mentioned	Kang et al. (2024)
Compressive strength: ECM/PCL (0.58 ± 0.02 MPa) and MD/PCL (0.43 ± 0.01 MPa)	Gradual Mg^2+^ ion release over 12 weeks; rapid in first 4 weeks	In water bath	Not explicitly mentioned	Li et al. (2023a)
Compressive modulus of Alg-nHAP: 0.007 ± 0.0002 MPa; higher in Alg-nHAP/CS-HA	51.58% degradation over 15 weeks in PBS	In an incubator	10.24-fold increase in swelling over 10 h	Banihashemian et al. (2024)
nHCA had highest tensile and compressive modulus compared to others	Not explicitly mentioned	In an incubator	Not explicitly mentioned	Zheng et al. (2014)
Compression modulus of SF-MMT: 24.78 ± 4.13 kPa; improved viscoelastic properties	Gradual degradation over 91 days in PBS	In an incubator	Higher swelling ratio than SF alone	Sheng et al. (2022)
Compression modulus of nHAMA scaffolds was three times higher than control	Not explicitly mentioned	In an incubator	Not explicitly mentioned	Zheng et al. (2023)
Improved compressive strength of GelMA with IGF-1bsn incorporation	Gradual degradation over 12 weeks *in vivo*	In an incubator	Not explicitly mentioned	Wu et al. (2023)
Improved compressive strength with dual crosslinking; stiffness increased	Slower degradation with DCM/DBM; sustained exosome release over 24 days	In an incubator	Improved swelling with DCM/DBM	Li et al. (2023b)
Compression modulus increased with HA gradient; Young’s modulus correlated with MagHA content	Gradual degradation; slower with higher MagHA content	In an incubator	Increased swelling with MagHA; faster equilibrium	Zhang et al. (2023a)
Compressive modulus: SS (subchondral) ∼ 100.09 ± 5.46 kPa, SC (cartilage) ∼ 50.2 ± 1.31 kPa	Not explicitly mentioned	In an incubator	SC hydrogel: 53.15%; SS hydrogel: 47.85%	You et al. (2018)
Compressive modulus of PCL/HA scaffolds: 14.86 ± 1.81 MPa; enhanced mechanical strength	GelMA hydrogel degraded rapidly; PCL/HA stable over 35 days	In an incubator	GelMA hydrogels showed rapid swelling	Zhang et al. (2023b)
Compressive modulus at interfacial region: 930 Pa; increased elastic modulus	Gradual degradation *in vivo*; complete defect closure after 8 weeks	In an incubator	Not explicitly mentioned	Radhakrishnan et al. (2018)
Compressive strength of gradient scaffold (G-nHA) ∼900 kPa; tensile strength improved	Gradual degradation over 28 days in PBS	In an incubator	Swelling equilibrium in 7 h; ratio of 6	Zhang et al. (2021)
Compression modulus decreased by 34.5% in TCP-loaded scaffolds; stable viscoelastic properties	Not explicitly mentioned	In an incubator	Swelling reduced by 18% in TCP-loaded scaffolds	Kosik-Kozioł et al. (2019)
Compression modulus enhanced by host-guest interactions; resilient and injectable	Gradual degradation over 28 days	In an incubator	Higher swelling ratio than GelMA hydrogels	Xu et al. (2019)
Compressive modulus: bone region: 1.95 ± 0.08 MPa; cartilage: 0.85 ± 0.11 MPa	Not explicitly mentioned	In an incubator	Cartilage region showed high liquid uptake	Qin et al. (2020)
Compressive strength: LDH scaffolds: 0.43 MPa; increased to 0.48 MPa with LL37 modification	Gradual degradation *in vivo* after 12 weeks	In an incubator	Not explicitly mentioned	Liu et al. (2021b)
Compressive modulus: 0.21–0.53 MPa; optimal scaffolds similar to natural cartilage	Gradual degradation over 8 weeks in lysozyme	In an incubator	Water absorption varied with composition	Hu et al. (2022)
Compressive modulus: 12 kPa (top layer) to 76 kPa (bottom layer)	Gradual degradation observed over 8 weeks	In an incubator	Not explicitly mentioned	Li et al. (2022)
Compressive modulus: CRH (62.7 kPa), BRH (56.8 kPa); improved with β-CD integration	Gradual degradation over 36 days in simulated joint environment	In an incubator	Not explicitly mentioned	Liu et al. (2020)
Compression strength increased with HA concentration; 27 kPa (non-mineralized) to 380 kPa (highly mineralized)	Gradual degradation observed over 28 days	In an incubator	Not explicitly mentioned	Fan et al. (2021)
Compressive modulus increased from 0.15 MPa to 0.58 MPa with 5% MgSO_4_	Not explicitly mentioned	In an incubator	Swelling rate reduced from 155% to 75%	Luo et al. (2022)
Compressive strength: 0.70 MPa; enhanced properties due to PDA and HA	GelMA-PDA/HA hydrogels degraded in 19 days	In an incubator	Low swelling ratio of 180%, minimal distortion	Gan et al. (2019)
Compression modulus increased by 61% with 60 wt% nHA; ultimate strength increased by 87%	Gradual sustained degradation allowing bioactive factor release over 21 days	In an incubator	Not explicitly mentioned	Castro et al. (2015)
Compressive strength: Virgin scaffolds (142 ± 14 MPa), Composite (62 ± 6 MPa), Osteochondral (85 ± 5 MPa)	Not explicitly mentioned	In an incubator	Not explicitly mentioned	Shalumon et al. (2016)
Compressive strength of HAp@PLL scaffold; mechanical strength sustained throughout regeneration	Hydrogel maintained hypoxic microenvironment for up to 20 days	In an incubator	Not explicitly mentioned	Guo et al. (2024)
Improved mechanical properties with Eu-HA nanorods in GelMA hydrogel	Gradual degradation in Eu-HA nanocomposite hydrogel	In an incubator	Not explicitly mentioned	Jin et al. (2024)

BRH, bone regenerating hydrogel; CRH, cartilage-regenerating hydrogel; EU-HA, Europium-doped Hydroxyapatite; PBS, Phosphate-Buffered Saline; PDA, polydopamine.

Biomimetic designs incorporating GelMA and HA have shown promise in enhancing mechanical stability and bioactivity for bone regeneration applications. GelMA hydrogels, while beneficial for tissue engineering, lack sufficient mechanical strength and osteogenic factors (Wang et al., 2022a). Incorporating HA into GelMA hydrogels improves their mechanical properties, biocompatibility, and osteogenic potential (Suvarnapathaki et al., 2020). Mineralized HA nanofibers further enhance the mechanical and bone regenerative performances of GelMA composites (Wang et al., 2022a). GelMA-based biomaterials can be tailored to overcome challenges in bone tissue engineering, such as insufficient mechanical properties and uncontrolled degradation (Dong et al., 2019). Advanced designs combining GelMA with other materials, like methacrylated HA nanoparticles and l-arginine-based unsaturated poly (ester amide), can create periosteum-mimicking scaffolds with improved mechanical strength, tissue adhesion, and osteogenic-angiogenic coupling effects (Yang et al., 2021). Double-crosslinking and freeze-drying methods have also been widely applied, producing physically and chemically reinforced structures that retain mechanical properties under physiological conditions (Yan et al., 2015; Filová et al., 2020; Zheng et al., 2023).

Balancing degradation rates with tissue regeneration remains another core challenge. An ideal scaffold degrades gradually, transferring mechanical load to newly forming tissue to aid integration (Hu et al., 2022; Li et al., 2022; Banihashemian et al., 2024; Chen et al., 2024). Studies have shown that adjusting crosslinking density and introducing bioactive molecules can customize degradation profiles for specific applications (Radhakrishnan et al., 2018; Zhang et al., 2023a; Zhang et al., 2023b; Deng et al., 2024). For example, Chen et al. developed a trilayered hydrogel with varied degradation rates across layers to replicate the native tissue gradient from cartilage to bone, facilitating sustained cell infiltration and extracellular matrix formation (Chen et al., 2024). Recent research has focused on developing multilayered hydrogel scaffolds to mimic the zonal organization of native cartilage tissue. These scaffolds feature gradients in mechanical properties, extracellular matrix composition, and bioactive factors across layers to guide cell differentiation and tissue formation (Brady et al., 2017; Qiao et al., 2021). Furthermore, a study demonstrated that layer-specific biomaterial compositions could direct a single stem cell population into zone-specific chondrocytes, resulting in native-like cartilage with varying mechanical and biochemical properties (Nguyen et al., 2011). In addition, a study further showed that stiffness gradient hydrogels could induce zone-specific responses in both chondrocytes and mesenchymal stem cells, mimicking cartilage zonal organization (Zhu et al., 2018). These approaches offer promising strategies for engineering complex osteochondral tissues with spatially-varying properties that more closely resemble native tissue structure and function.

Future advancements will likely focus on refining crosslinking techniques, such as enzyme-catalyzed, thermal, and photo-crosslinking, to develop materials that meet both mechanical and degradation needs for effective tissue engineering.

### 3.4 Biocompatibility and functional characteristics

Nano-hydrogel systems have consistently demonstrated excellent biocompatibility and functional characteristics, making them highly suitable for applications in tissue engineering, particularly in osteochondral regeneration. Studies have reported cell viability rates exceeding 90% and enhanced cell proliferation, supporting the potential of these materials to promote tissue growth and regeneration (Table 3). For example, a study showed that LiMn_2_O_4_ nanozyme-functionalized hydrogels effectively supported the proliferation of rat chondrocytes and bone marrow-derived mesenchymal stem cells (BMSCs), promoting cell adhesion and growth (Hu et al., 2024). In addition, *in vitro* studies have highlighted that nano-hydrogels, such as GH@LM + GA@HLM and Zn-AlgMA, significantly enhance the proliferation of both chondrocytes and BMSCs, while maintaining high levels of cell viability (Hu et al., 2024; Zhang et al., 2024). Similarly, functionalized scaffolds, including those with CK2.1/LL37 and SF-MMT, further promote the regenerative processes of BMSCs and chondrocytes, reinforcing the critical role of scaffold composition in optimizing cellular responses (Liu et al., 2021b; Sheng et al., 2022).

**TABLE 3:** Biocompatibility and functional characteristics.

Cell types used	Culture conditions	Viability and proliferation	Bioactivity	Functionalization and targeting	References
BMSCs, chondrocytes	DMEM with 10% FBS, 1% Penicillin/Streptomycin; osteogenic and inflammatory induction	>90% viability; enhanced proliferation in CuTA@SF	Promoted osteogenesis and chondrogenesis	Targeted osteochondral regeneration, cartilage and bone repair	Cao et al. (2023)
MC3T3-E1 cells, chondrocytes	Media leached from hydrogel layers over 7 days	>90% viability; enhanced proliferation for both cell types	Promoted osteogenesis and chondrogenesis	Targeted osteochondral regeneration	Lan et al. (2021)
L929 fibroblasts, C3H mouse MSCs, mouse chondrocytes	DMEM/F12 with IL-4; osteogenic induction media for MSCs	>97% viability; no significant difference in growth	Promoted anti-inflammatory effects, and chondrogenesis	Targeted osteochondral regeneration	Gong et al. (2020)
rBMSCs	Cultured with KGN and AT in induction media for 14 days	>95% viability; good proliferation confirmed	Enhanced chondrogenesis and osteogenesis	Targeted for osteochondral regeneration	Chen et al. (2024)
Rat chondrocytes, BMSCs	Treated with ROS inducer H_2_O_2_	>95% viability; high proliferation	Enhanced chondrogenesis and osteogenesis	Designed for osteochondral repair	Hu et al. (2024)
rBMSCs	Cultured in basal and osteogenic media for up to 14 days	>90% viability; increase in proliferation over 14 days	Enhanced osteogenesis in silk-nanoCaP layer	Targeted osteochondral repair with distinct layers	Yan et al. (2015)
MC3T3-E1 preosteoblasts, ATDC5 chondrocytes	Co-cultured in layers for 7 days, then 21 days	>85% viability; significant increase in metabolic activity	Enhanced chondrogenesis (COL II) and osteogenesis (COL I, ALP)	Designed for osteochondral repair	Korpayev et al. (2020)
BMSCs	Cultured in thermogel layer with KGN	High viability maintained	Enhanced chondrogenesis and osteogenesis	Full-thickness osteochondral repair	Zhang et al. (2022b)
WRN cells	Encapsulated in hydrogels with Fe_3_O_4_ nanoparticles for 48 h	High viability; no cytotoxicity	Fe_3_O_4_ nanoparticles exert physiological forces on encapsulated cells	Injectable scaffolds for osteochondral regeneration	Adedoyin et al. (2015)
BMSCs	Cultured in osteogenic and chondrogenic media with immersion liquid	>90% viability; proliferation in Zn-AlgMA hydrogel at 10^−4^ M zinc ion	Enhanced osteogenesis (Mg^2+^) and chondrogenesis (Zn^2+^)	Targeted osteochondral repair	Zhang et al. (2024)
rBMSCs and chondrocytes	Cultured on GelMA and DE-incorporated scaffolds in induction media	High cell viability observed on 5%–20% DE scaffolds	DE microparticles significantly enhanced chondrocyte proliferation	Dual-layer scaffolds for cartilage and bone regeneration	Deng et al. (2024)
Fibrochondrocytes, chondrocytes	Cultured on PCL-chitosan and anti-CD44-modified microparticles	High viability	Anti-CD44 microparticles enhanced osteogenic regeneration	Targeted osteochondral defects	Filová et al. (2020)
Porcine chondrocytes	Encapsulated in PEGDA-DTT hydrogels for 7 days	>95% viability	Heparin promoted sustained release and enhanced differentiation	Craniofacial reconstruction, supporting cartilage and bone	Brown et al. (2024)
hBMSCs	Cultured in scaffolds with DMEM and supplements	High viability confirmed	Enhanced chondrogenesis and osteogenesis	Targeted osteochondral defect repair	Liu et al. (2021a)
MSCs and chondrocytes	Cultured in hydrogel scaffolds	High viability confirmed	Enhanced chondrogenesis and osteogenesis	Targeted osteochondral regeneration	Kang et al. (2024)
hBMSCs	Cultured on ECM/PCL and MD/PCL scaffolds	High viability confirmed	ECM/PCL promoted huBMSC proliferation	Targeted osteochondral defects	Li et al. (2023a)
hCHCs and hAdMSCs	Cultured in CS-HA and Alg-nHAP scaffolds	High viability	Significant proliferation in both scaffold types	Targeted osteochondral repair	Banihashemian et al. (2024)
Chondrocytes from newborn rabbit	Encapsulated in nHCA, HCA, and nHC hydrogels for 21 days	High viability	nHCA showed highest cell proliferation	Targeted osteochondral regeneration	Zheng et al. (2014)
BMSCs and chondrocytes	Cultured in SF-MMT and SF with osteogenic induction	>93% viability	Increased proliferation with no significant difference	Targeted osteochondral regeneration	Sheng et al. (2022)
ADSCs	Cultured in nHAp and nHApMA bio-inks	High viability confirmed	Enhanced osteogenic and chondrogenic differentiation	Targeted osteochondral regeneration	Zheng et al. (2023)
rBMSCs	Cultured in GelMA and GelMA/IGF-1bsn hydrogels for 72 h	High viability confirmed	BSN-GelMA significantly enhanced rBMSC proliferation	Osteochondral regeneration in mosaicplasty	Wu et al. (2023)
rBMSCs	Cultured in Hydrogel-DCM and Hydrogel-DBM for 14 days	High viability confirmed	Exosome-loaded scaffolds enhanced proliferation	Targeted osteochondral repair	Li et al. (2023b)
BMSCs	Cultured in MagHA-gradient hydrogel for 21 days	High viability confirmed	Significant proliferation in MagHA gradient compared to control	Full-thickness osteochondral regeneration	Zhang et al. (2023a)
rBMSCs	Encapsulated in SC and SS hydrogels	>90% viability	Significant proliferation in both hydrogels	Designed for osteochondral regeneration	You et al. (2018)
BMSCs	Cultured in KGN-loaded GelMA and HA-coated PCL scaffolds	High viability confirmed	Significant proliferation in both cartilage and bone regions	Targeted osteochondral repair	Zhang et al. (2023b)
Rat osteoblasts and caprine chondrocytes	Co-cultured in gradient hydrogel for 21 days	High viability confirmed	Higher proliferation in nHA-enriched hydrogels	Designed for osteochondral regeneration	Radhakrishnan et al. (2018)
Goat TMJ disc cells	Cultured in nHA-gradient hydrogels; assessed via MTT and AO/EB staining	High viability confirmed	Increased proliferation in G-nHA scaffold compared to controls	Targeting cartilage and subchondral bone with gradient layers	Zhang et al. (2021)
BM-hMSCs	Cultured in chondrogenic media for 21 days	High viability confirmed	Increased proliferation in TCP-loaded scaffolds	Designed for calcified cartilage and subchondral bone regeneration	Kosik-Kozioł et al. (2019)
hBMSCs	Encapsulated in HGM and GelMA hydrogels with TGF-β1 or KGN for 14 days	>95% viability	Significant proliferation in HGM compared to GelMA	Injectable for osteochondral regeneration	Xu et al. (2019)
hASCs	Seeded into bilayer scaffold with BMP-2 and IGF-1 for 14 days	High viability observed	Cells proliferated and formed spheroids in cartilage region	Sequential chondrogenesis and osteogenesis mimicking natural tissue	Qin et al. (2020)
MSCs and HUVECs	Cultured in CK2.1/LL37-loaded scaffolds for 14 days	High viability observed	Enhanced proliferation in CK2.1/LL37 scaffolds	Targeting cartilage and subchondral bone	Liu et al. (2021b)
ADSCs	Cultured in multilayer scaffolds in static and dynamic environments	>90% viability	Higher proliferation in dynamic culture compared to static	Layered design for cartilage and subchondral bone targeting	Hu et al. (2022)
BMSCs	Cultured in multi-layer scaffold in osteogenic and chondrogenic media	>95% viability	Significant proliferation in both regions	Targeting cartilage and subchondral bone in distinct layers	Li et al. (2022)
hMSCs	Encapsulated in CRH and BRH hydrogels for 21 days	>90% viability	Significant proliferation with phase-specific differentiation	Simultaneous regeneration of cartilage and subchondral bone	Liu et al. (2020)
BMSCs	Cultured in gradient mineralized hydrogels for 21 days	>95% viability	Good proliferation in non-mineralized and mineralized layers	Mimicking cartilage and subchondral bone regions with gradients	Fan et al. (2021)
BMSCs	Cultured in Mg^2+^- and Cu^2+^-regulated layers	High viability observed	Enhanced proliferation in regulated hydrogels	Designed for osteochondral regeneration	Luo et al. (2022)
BMSCs and chondrocytes	Cultured on GelMA, GelMA-PDA, and GelMA-PDA/HA	High viability confirmed	Significant proliferation in PDA-incorporated hydrogels	Targeting cartilage and subchondral bone in dual-layer structure	Gan et al. (2019)
hMSCs	Cultured on PEG-DA scaffolds with nHA and TGF-β1	High viability; significant proliferation observed	93% and 53% increase for 40 wt% and 60 wt% nHA	Designed for osteochondral regeneration	Castro et al. (2015)
BMSCs and chondrocytes	BMSCs in osteogenic medium, chondrocytes in chondrogenic medium	>90% viability	Significant proliferation in both parts	Designed for osteochondral tissue engineering	Shalumon et al. (2016)
BMSCs and chondrocytes	Encapsulated in GelMA microspheres in induction media	>90% viability	Significant proliferation; enhanced differentiation confirmed	Targeting cartilage and subchondral bone for complex regeneration	Guo et al. (2024)
Chondrocytes, BMSCs, RAW264.7 macrophages	Cultured in DMEM/F12, α-MEM, and DMEM with 10% FBS	>90% viability	Promotion of chondrocyte proliferation and BMSC differentiation	Designed to facilitate immunomodulation for osteochondral regeneration	Jin et al. (2024)

BMSCs, Bone Marrow Mesenchymal Stem Cells; hBMSCs, Human Bone Marrow Mesenchymal Stem Cells; rBMSCs, rabbit Bone Marrow Mesenchymal Stem Cells; BM-hMSCs, Bone Marrow-Derived Human Mesenchymal Stem Cells; hMSCs, Human Mesenchymal Stem Cells; TMJ, temporomandibular joint; hCHCs, Human Chondrocyte-like Cells; hAdMSCs, Human Adipose-derived Mesenchymal Stem Cells; WRN, wnt rspondin noggin cells; hASCs, Human adipose-derived stem cells.

Nano-hydrogels mimicking the extracellular matrix (ECM) have emerged as promising scaffolds for tissue engineering and regenerative medicine. These biomimetic materials create a three-dimensional (3D) environment that closely resembles the native ECM’s nanoscale architecture (Geckil et al., 2010; Gough et al., 2012; Brown et al., 2024). By incorporating nanostructured components, such as nanofibers or nanosilicates, these hydrogels can actively modulate cellular responses, including attachment, proliferation, and differentiation (Wei and Ma, 2008). For instance, nanoengineered collagen-based hydrogels reinforced with disk-shaped nanosilicates have been shown to enhance osteogenic differentiation of human mesenchymal stem cells without the need for exogenous growth factors (Paul et al., 2016). These ECM-mimicking hydrogels not only provide structural support but also create a regulatory milieu that guides tissue formation and organization (Geckil et al., 2010). Furthermore, their biocompatibility and ability to induce regenerative processes make them promising candidates for various biomedical applications, including bone tissue engineering and *in vitro* disease modeling (Wei and Ma, 2008; Paul et al., 2016).

Furthermore, functionalization techniques are crucial for enhancing the bioactivity of hydrogels in osteochondral tissue engineering. By incorporating growth factors, bioactive molecules, and nanoparticles, these hydrogels can promote both osteogenesis and chondrogenesis. For example, research has shown that embedding polydopamine-encapsulated kartogenin (KGN) and calcium phosphate-encapsulated miRNA-26a within hydrogels effectively promotes regeneration in both cartilage and bone layers (Kang et al., 2024). Additionally, KGN has been grafted onto ultrasmall superparamagnetic iron-oxide nanoparticles, which are then integrated into hydrogels for cartilage repair while enhancing MRI contrast (Yang et al., 2019). Another study developed microscaffold-hydrogel composites containing KGN and peptides to accelerate osteochondral repair through endochondral ossification (Zhang et al., 2022a). Moreover, a versatile hydrogel system using click chemistry has been created to provide tissue-specific cues for either chondrogenesis or osteogenesis (You et al., 2018; Guo et al., 2020; Liu et al., 2021a; Li et al., 2023a). These approaches highlight the potential of functionalized hydrogels in addressing the complex requirements of osteochondral tissue regeneration.

Recent studies demonstrate the effectiveness of functionalized biomaterials in advancing osteochondral repair, primarily by supporting both osteogenic and chondrogenic differentiation. Composite hydrogels with anti-CD44-labeled microparticles have shown to significantly improve osteogenic regeneration in animal models of osteochondral defects (Filová et al., 2020). Likewise, bilayer scaffolds that guide stem cell differentiation spatially have been effective in directing cells into osteogenic and chondrogenic lineages, enhancing repair outcome (Kang et al., 2024; Lowen et al., 2024). Furthermore, microscaffold-hydrogel composites, incorporating bioactive modifications like RGD peptides, have demonstrated accelerated osteochondral repair through endochondral ossification, achieved by controlled delivery of bioactive molecules within the scaffold layers (Zhang et al., 2022a; Brown et al., 2024; Deng et al., 2024). Other studies reinforce these findings, with functionalized hydrogels designed for dual osteogenic and chondrogenic applications showing sustained, layer-specific release of growth factors and bioactive ions, thus promoting cell proliferation and tissue integration (Cao et al., 2023; Wu et al., 2023).

These findings underscore the potential of multi-functionalized nano-hydrogels in tissue engineering, with customizable layers enabling the spatially controlled release of bioactive agents that foster site-specific tissue regeneration. Such approaches pave the way for advanced therapies for osteochondral defects and other complex tissue engineering applications (Wu et al., 2023; Brown et al., 2024).

These findings suggest that nano-hydrogels are capable of providing a supportive 3D microenvironment that mimics the native ECM. However, achieving consistent differentiation and integration remains challenging, particularly when translating *in vitro* success to *in vivo* conditions. Variability in cell behavior across studies suggests that more standardized protocols are needed to optimize cell-scaffold interactions, ensuring predictable outcomes in clinical settings.

### 3.5 *In vivo* efficacy and regeneration outcomes

The *in vivo* studies summarized in Table 4 illustrate the promising efficacy of nano-hydrogels in promoting osteochondral repair, using diverse animal models such as rabbits, rats, and mice to assess the regenerative potential of these systems. Significant cartilage regeneration and subchondral bone repair were observed in a rabbit model using a bi-layered GelMA-PCL-HA scaffold, where histological analyses confirmed the formation of a smooth cartilage surface and well-integrated bone layer (Gong et al., 2020). Similarly, a bilayer hydrogel containing GH@LM + GA@HLM demonstrated notable regeneration, with micro-CT and histological assessments indicating smooth hyaline cartilage formation and robust subchondral bone repair (Hu et al., 2024) (Table 4). These advanced hydrogel systems have demonstrated improvements in defect filling, cartilage thickness, and bone regeneration compared to control groups (Gan et al., 2019; Guo et al., 2021). However, a critical review of *in vivo* cartilage repair studies highlights the need for standardized experimental designs and careful interpretation of results (Vilela et al., 2015).

**TABLE 4:** Experimental models and methods *in vivo* studies.

Animal model	Group allocation	Implantation method	Histological assessment	References
Rabbits	5 groups: Control, SF, Cu@SF, TA@SF, CuTA@SF	Pre-formed hydrogels implanted into OCD site	CuTA@SF showed the best integration and cartilage repair	Cao et al. (2023)
Rabbits	3 groups: Blank, PVA hydrogel, Bi-layer hydrogel	Hydrogels implanted into defects created in rabbit knees	Bi-layer group showed better cartilage and bone repair	Lan et al. (2021)
Rabbits	3 groups: Nontreated, bi-layer scaffold, and IL-4-loaded bi-layer scaffold; 8- and 16-week post-surgery observations	Bi-layer scaffold implanted into defects created in rabbit knee joints	IL-4-loaded scaffold group showed better cartilage repair	Gong et al. (2020)
Rabbits	3 groups: Untreated (blank), control, experimental	Trilayered scaffolds implanted into osteochondral defects	Experimental group showed better cartilage and bone repair	Chen et al. (2024)
Sprague-Dawley rats	4 groups: PBS, GH + GA (basic hydrogel), GH + GA@H (with nanohydroxyapatite), GH@LM + GA@HLM (with nanozyme)	Bilayer hydrogels implanted into femoral condyle defects	GH@LM + GA@HLM showed the best cartilage and subchondral bone repair	Hu et al. (2024)
Rabbits	2 groups: bilayered scaffold implantation and defect control (no scaffold)	Bilayered scaffolds were press-fit into osteochondral defects in rabbit knees	Scaffold showed cartilage and subchondral bone regeneration	Yan et al. (2015)
BALB/c mice	Specific details are not explicitly mentioned	Multi-layered scaffolds were inserted into subcutaneous pockets created in mice	Staining showed mild inflammatory response with macrophage and neutrophil infiltration	Korpayev et al. (2020)
Rabbits	4 groups: control, Gel/Scaffold, Gel-MSCs/Scaffold, GelKGN-MSCs/ScaffoldBMP-2	Bilayered scaffolds were implanted into osteochondral defects in the femoral condyle	Staining showed cartilage and subchondral bone regeneration in the GelKGN-MSCs/ScaffoldBMP-2 group	Zhang et al. (2022b)
Rabbits	4 groups: blank control, Zn-AlgMA, DCPD-coated Mg, Zn-AlgMA@Mg scaffold	Scaffolds implanted into osteochondral defects in femoral condyles	Zn-AlgMA@Mg group showed best osteochondral integration	Zhang et al. (2024)
Rabbits	4 groups: blank control, GelMA, 0–10 DE, 5–20 DE scaffolds	Scaffolds implanted in femoral condyle defects	5–20 DE group showed best osteochondral regeneration	Deng et al. (2024)
Rabbits	3 groups: scaffold #1 (PCL-chit-PEGb), scaffold #2 (PCL-chit-PEGb-antiCD44), control	Scaffolds implanted in femoral condyle defects	PCL-chit-PEGb showed superior hyaline cartilage regeneration, while anti-CD44 favored bone formation	Filová et al. (2020)
Rabbits	3 groups: Control, single-layer scaffold, bilayer scaffold	Bilayer scaffolds implanted into knee joint defects	Bilayer scaffold showed better cartilage regeneration and bone formation	Liu et al. (2021a)
Rabbits	5 groups: Control, GTU-Fe, GTU-Fe/KGN@PDA, GTU-Fe/miRNA@CaP, GTU-Fe/KGN@PDA/miRNA@CaP	Cylindrical GTU-Fe scaffolds implanted into knee defects	GTU-Fe/KGN@PDA/miRNA@CaP showed better cartilage and bone regeneration	Kang et al. (2024)
Rats	5 groups: Blank, PCL, ECM/PCL, MD/PCL, Bilayer scaffold	Bilayer scaffolds implanted into knee joint defects	Bilayer scaffold showed better cartilage and bone regeneration	Li et al. (2023a)
Rabbits	3 groups: Control, SF hydrogel, SF-MMT hydrogel	SF and SF-MMT hydrogels implanted into osteochondral defects in rabbit knees	SF-MMT showed better cartilage and bone regeneration	Sheng et al. (2022)
Rabbits	3 groups: Control, nHAp bio-ink, nHApMA bio-ink	Scaffolds implanted into femoral condyle defects in rabbit knees	nHApMA showed better cartilage and bone regeneration	Zheng et al. (2023)
Rabbits	3 groups: Blank, GelMA hydrogel, BSN-GelMA hydrogel	Mosaicplasty performed on rabbit knee joints	BSN-GelMA showed better gap integration and tissue regeneration	Wu et al. (2023)
Rats	4 groups: Blank, Hydrogel, Bi-Hydrogel, Bi-Hydrogel-Exos	Bilayer scaffolds implanted into osteochondral defects in rat knee joints	Bi-Hydrogel-Exos showed better osteochondral regeneration	Li et al. (2023b)
Rabbits	5 groups: Control, DN hydrogel, bi-phasic hydrogel, MagHA gradient hydrogel with (Gra+) and without (Gra-) magnetic field stimulation	Hydrogel scaffolds implanted into rabbit knee joint defects	MagHA-gradient hydrogel showed enhanced osteochondral regeneration, especially in Gra+	Zhang et al. (2023a)
C57BL/6J mice	Specific details are not explicitly mentioned	SC and SS hydrogels implanted under dorsal skin	Staining showed good integration of hydrogels with surrounding tissue	You et al. (2018)
Rabbits	4 groups: Blank, PCL/GelMA, PCL/GelMA@TA/E7, PCL/HA-GelMA/KGN@TA/E7	Bilayer scaffolds implanted into knee joint defects	PCL/HA-GelMA/KGN@TA/E7 group showed better cartilage and subchondral bone regeneration	Zhang et al. (2023b)
Rabbits	4 groups: Control, nHA scaffold, ChS scaffold, Gradient (nHA + ChS) scaffold	Hydrogels injected into osteochondral defects in rabbit knees	Gradient scaffold group showed improved collagen and GAG deposition	Radhakrishnan et al. (2018)
Rats	7 groups: Control, BMSCs only, 0% nHA + BMSCs, 40% nHA + BMSCs, 70% nHA + BMSCs, G-nHA only, G-nHA + BMSCs	Scaffolds implanted into rat knee defects	G-nHA + BMSCs group showed better osteochondral regeneration	Zhang et al. (2021)
Rats	4 groups: GelMA with KGN, GelMA with TGF-β1, HGM (Injection) with KGN, HGM (Injection) with TGF-β1	HGM hydrogels injected into defects in rat knees	HGM groups showed better cartilage and subchondral bone regeneration	Xu et al. (2019)
Rabbits	3 groups: Control, biphasic scaffold without peptide (GC/LC), biphasic scaffold with CK2.1/LL37 (CK2.1@GC/LL37@LC)	Scaffolds implanted into osteochondral defects	CK2.1/LL37 group showed better cartilage and subchondral bone regeneration	Liu et al. (2021b)
Rabbits	4 groups: Negative control, positive control, static scaffold group, dynamic scaffold group	Multilayer scaffolds implanted into knee defects in rabbits	Dynamic scaffold showed better osteochondral regeneration compared to the static group	Hu et al. (2022)
Rabbits	3 groups: Control, GelMA scaffold, nHA-GelMA scaffold	Scaffolds implanted into osteochondral defects in rabbit knee joints	nHA-GelMA showed better osteochondral regeneration	Li et al. (2022)
Rabbits	4 groups: Control, Drug-free BRH-CRH, BRH-CRH (no MSCs), BRH-CRH [MSC-encapsulated]	Bilayer BRH-CRH hydrogel scaffolds injected osteochondral defect site	BRH-CRH [MSC-encapsulated] showed better osteochondral integration and cartilage regeneration	Liu et al. (2020)
Rabbits	3 groups: Control, DN material group, DN-3Mg/Cu hydrogel group	Bilayer hydrogels implanted into osteochondral defects in rabbits	DN-3Mg/Cu hydrogel showed better osteochondral regeneration	Luo et al. (2022)
Rabbits	3 groups: Pure GelMA, bilayer GelMA-PDA/HA, bilayer GelMA-PDA/HA with BMP-2 and TGF-β3	Bilayer hydrogels implanted into osteochondral defects in rabbit knee joints	BMP-2/TGF-β3 showed well-organized cartilage and subchondral bone regeneration	Gan et al. (2019)
Nude mice	2 groups: Acellular scaffold and cell-seeded scaffold (sample)	Cell-seeded scaffolds implanted subcutaneously in nude mice	Staining confirmed tissue-specific regeneration of bone and cartilage in scaffolds	Shalumon et al. (2016)
Rabbits	4 groups: HAp@PLL scaffold, Ta@gel + GelMA@BMSCs, HAp@PLL + hydrogel + GelMA@BMSCs, and HAp@PLL + Ta@gel + GelMA@BMSCs	Composite scaffolds were implanted in 4 mm osteochondral defects in rabbit knee joints	HAp@PLL + Ta@gel + GelMA@BMSCs revealed better osteochondral regeneration	Guo et al. (2024)
Rats	4 groups: Control, GelMA, GelMA/HAp, and GelMA/Eu-HAp	GelMA/Eu-HAp hydrogel was injected into osteochondral defects	GelMA/Eu-HAp showed better cartilage and bone regeneration	Jin et al. (2024)

Immunohistochemistry	Inflammation and infection	Degradation of hydrogel	References
Strong positive staining for COL I and AGG in both cartilage and subchondral bone regions in CuTA@SF group	No signs of infection	CuTA@SF degraded almost completely by week 12 than other groups	Cao et al. (2023)
Positive staining for COL I and COL II in cartilage and subchondral bone regions, indicating successful tissue repair	No signs of infection	Upper layer degraded faster than lower; neither layer completely degraded after 12 weeks	Lan et al. (2021)
Positive COL2 staining in IL-4 scaffold group, indicating cartilage regeneration	No signs of infection	GelMA layer showed gradual degradation over 16 weeks	Gong et al. (2020)
COL2 and OCN staining showed significant matrix deposition in cartilage and bone regions in the experimental group	No signs of infection	Gradual degradation of scaffolds over 12 weeks; KGN and AT released during scaffold degradation	Chen et al. (2024)
Strong COL II and Aggrecan in cartilage, COL I and Opn in bone for GH@LM + GA@HLM group	No signs of infection	GH@LM + GA@HLM hydrogel gradually degraded over 12 weeks	Hu et al. (2024)
Positive staining for collagen II in cartilage and new bone formation in the silk-nanoCaP layer	No signs of infection	Scaffold maintained integrity with no significant mass loss over 4 weeks	Yan et al. (2015)
Not reported	Inflammation noted at the scaffold interface	Not explicitly mentioned	Korpayev et al. (2020)
Positive staining for COL II in cartilage and COL I in subchondral bone confirmed tissue regeneration	Minimal inflammation observed	Scaffold and thermogel gradually degraded over 3–6 months	Zhang et al. (2022b)
Positive COL II staining in cartilage and COL I staining in bone confirmed tissue regeneration	No signs of infection	Gradual degradation of the Zn-AlgMA hydrogel and Mg alloy over the course of the 10-week study	Zhang et al. (2024)
Positive COL II and COL I staining confirmed cartilage and bone tissue regeneration in 5–20 DE scaffolds	No signs of infection	Slower degradation observed in DE-incorporated scaffolds, with 5–20 DE showing the slowest rate	Deng et al. (2024)
COL II and osteocalcin revealed scaffold #1 (PCL-chit-PEGb) promoted cartilage, while scaffold #2 (anti-CD44) favored bone formation	anti-CD44 exhibited more inflammatory infiltration	Not explicitly mentioned	Filová et al. (2020)
Positive staining for COL I and COL II confirmed cartilage and bone tissue regeneration	No signs of infection	Not explicitly mentioned	Liu et al. (2021a)
Positive staining for COL II and COL I confirmed tissue regeneration	No signs of infection	Gradual degradation observed over 12 weeks post-implantation	Kang et al. (2024)
Positive staining for COL II in cartilage and COL I in subchondral bone confirmed tissue regeneration	No signs of infection	Gradual degradation of the scaffold over 12 weeks, with sustained Mg^2+^ release	Li et al. (2023a)
Positive staining for Aggrecan and COL II confirmed cartilage matrix formation in SF-MMT group	No signs of infection	Gradual degradation of the SF-MMT hydrogel over the course of the 12-week study	Sheng et al. (2022)
Positive staining for Runx2 and Sox9 confirmed osteogenic and chondrogenic differentiation in nHApMA scaffolds	No signs of infection	Not explicitly mentioned	Zheng et al. (2023)
Positive staining for collagen II confirmed enhanced cartilage regeneration in BSN-GelMA group	No signs of infection	Gradual degradation observed over 12 weeks	Wu et al. (2023)
Positive staining for COL II in cartilage and COL I in bone confirmed tissue regeneration	No significant inflammation observed in any group	Gradual degradation over 12 weeks, with good scaffold integration	Li et al. (2023b)
Positive staining for collagen II in cartilage and collagen I in bone confirmed tissue regeneration in MagHA groups	No signs of infection	Gradual degradation observed over 12 weeks; slower in MagHA-rich regions	Zhang et al. (2023a)
Not applicable	No signs of infection	Not explicitly mentioned	You et al. (2018)
Positive staining for COL II in cartilage and COL I in bone confirmed tissue regeneration	No signs of infection	Gradual degradation of GelMA observed over 12 weeks	Zhang et al. (2023b)
Not reported	No signs of infection	Gradual degradation observed over 8 weeks	Radhakrishnan et al. (2018)
Positive COL II staining confirmed cartilage regeneration in G-nHA + BMSCs group	No signs of infection	Gradual degradation of the scaffold over 12 weeks post-implantation	Zhang et al. (2021)
Positive staining for type II collagen confirmed chondrogenesis in HGM groups	No signs of infection	Gradual degradation of HGM hydrogels over 6 weeks	Xu et al. (2019)
Positive staining for COL I in subchondral bone and COL II in cartilage confirmed tissue regeneration	No signs of infection	Gradual degradation observed over 12 weeks post-implantation	Liu et al. (2021b)
Positive staining for COL II confirmed cartilage matrix formation in dynamic group	No signs of infection	Gradual degradation of the scaffold observed over 12 weeks	Hu et al. (2022)
Positive staining for COL II in cartilage and COL I in subchondral bone confirmed tissue regeneration	No signs of infection	Gradual degradation over 12 weeks	Li et al. (2022)
Positive staining for COL II in cartilage and COL I in bone confirmed phase-specific tissue regeneration	No signs of infection	Gradual degradation observed over 12 weeks *in vivo*	Liu et al. (2020)
Positive staining for type II collagen and GAG in cartilage, and collagen type I in bone in DN-3Mg/Cu group	No signs of infection	Gradual degradation observed over 12 weeks	Luo et al. (2022)
Not reported	No signs of infection	Gradual degradation over 12 weeks	Gan et al. (2019)
Positive staining for type I collagen (bone) and type II collagen (cartilage) confirmed osteochondral tissue formation	No signs of infection	Not explicitly mentioned	Shalumon et al. (2016)
Positive staining for COL II, ACAN, and SOX9 in cartilage, and COL I, OPN, and OCN in subchondral bone	No signs of infection	Gradual degradation of hydrogel observed, supporting tissue regeneration	Guo et al. (2024)
Positive staining for CD206 and Arg1 indicated M2 macrophage polarization in the GelMA/Eu-HAp group	No signs of infection	Gradual degradation of GelMA/Eu-HAp hydrogel observed over time	Jin et al. (2024)

Histological assessments across various studies frequently highlighted improved tissue integration. A GTU-Fe/KGN@PDA/miRNA@CaP scaffold led to enhanced chondrogenic and osteogenic marker expression, indicating successful differentiation and maturation of regenerated tissue, with elevated glycosaminoglycans (GAG) and collagen deposition contributing to effective cartilage and bone regeneration (Table 4) (Kang et al., 2024). Further corroborating these findings, a Zn-AlgMA@Mg scaffold achieved significant osteochondral integration, facilitating seamless cartilage repair and trabecular bone formation within femoral condyle defects in rabbits (Zhang et al., 2024) (Table 4). Despite these advancements, scaffold-cartilage integration remains a significant challenge in tissue engineering. Recent strategies to address this issue include manipulating cellular, material, and biomolecular composition of engineered tissue (Jelodari et al., 2022). These findings highlight the potential for improved cartilage repair and integration using advanced scaffolds and tissue engineering techniques.

Many studies achieved substantial subchondral bone regeneration, suggesting that functionalization strategies including the incorporation of miRNAs, bioactive molecules, and structurally adaptive hydrogels play a crucial role in promoting dual regeneration for osteochondral repair. For example, bi-layer hydrogels and trilayered scaffolds demonstrated enhanced bone volume and trabecular thickness, ultimately supporting comprehensive osteochondral regeneration (Lan et al., 2021; Chen et al., 2024). Moreover, these studies predominantly used femoral condyle defect models, effectively showing that nano-hydrogels, when tailored to recreate the native extracellular environment, support robust tissue regeneration over extended periods. Functionalization strategies, such as incorporating tissue-specific peptides or drugs, have shown enhanced chondrogenesis and osteogenesis both *in vitro* and *in vivo* (Guo et al., 2021; Chen et al., 2024). These advanced scaffolds have demonstrated improved bone volume, trabecular thickness, and overall defect filling in femoral condyle defect models, supporting comprehensive osteochondral regeneration (Cao et al., 2024; Chen et al., 2024; Guo et al., 2024).

The variability in regenerative outcomes observed across studies, characterized by differing degrees of bone density and cartilage smoothness, highlights the necessity for a standardized approach to evaluating scaffold performance. Future research should focus on adopting consistent animal models, such as femoral defect models, and harmonized assessment criteria, such as specific histological markers and imaging techniques, to enable comparative evaluations across various hydrogel systems. Such standardization could accelerate the translation of nano-hydrogel-based technologies into clinical settings, supporting more predictable outcomes and broader applicability.

### 3.6 Key limitations in osteochondral repair studies and prospective innovations

Recent advances in osteochondral tissue engineering have focused on developing scaffolds that support cell growth and tissue regeneration. Scaffold degradation plays a crucial role in the repair process, with different degradation modalities and speeds influencing outcomes (Tortorici et al., 2022). Despite considerable advances in osteochondral repair, several critical limitations remain across studies, as outlined in Table 5. One major challenge involves inconsistent degradation rates in scaffold materials. Achieving a uniform degradation timeline has proven difficult, with some hydrogel systems degrading faster than intended, reducing structural support for newly forming tissue, while others degrade too slowly, limiting cell infiltration and impeding tissue remodeling. For instance, study conducted by Adedoyin et al. noted this inconsistency in their dual-gelation scaffold, where uneven degradation impacted overall regenerative outcomes (Adedoyin et al., 2015). To address this, further research should investigate advanced crosslinking techniques to fine-tune degradation kinetics, ensuring scaffold resorption aligns more closely with native tissue growth.

**TABLE 5:** Summary of study limitations and proposed future directions.

Limitations	Future directions	References
No significant enhancement in mechanical properties; lack of long-term studies	Optimize CuTA concentration; conduct long-term *in vivo* studies	Cao et al. (2023)
Incomplete degradation after 12 weeks; mechanical properties do not match natural tissue	Optimize hydrogel composition; explore long-term repair outcomes	Lan et al. (2021)
Study limited to rabbits; need for investigation in larger animals or humans	Study IL-4 mechanisms in osteochondral repair in larger models	Gong et al. (2020)
Study limited to rabbits; larger animal models and longer-term studies needed	Investigate drug release mechanisms; test in larger animals	Chen et al. (2024)
Lack of biomechanical testing	Expand to larger models and conduct biomechanical tests	Hu et al. (2024)
Short-term study (4 weeks)	Investigate long-term effects; optimize mechanical properties	Yan et al. (2015)
Short-term *in vivo* study (14 days)	Conduct longer-term studies on scaffold degradation	Korpayev et al. (2020)
Short-term study	Optimize materials for cartilage and bone regeneration rates	Zhang et al. (2022b)
No *in vivo* testing conducted	Focus on *in vivo* testing for osteochondral repair	Adedoyin et al. (2015)
Short-term study (10 weeks)	Conduct long-term studies on degradation and integration	Zhang et al. (2024)
Short-term study (12 weeks)	Investigate long-term effects; optimize scaffolds for human use	Deng et al. (2024)
Short study duration; inflammatory response from scaffold #2	Assess long-term effects and optimize modifications to reduce inflammation	Filová et al. (2020)
Short-term study; no *in vivo* testing	Explore *in vivo* testing and growth factor delivery	Brown et al. (2024)
Short-term study	Focus on long-term integration and clinical translation	Liu et al. (2021a)
Short-term study	Optimize KGN and miRNA-26a delivery for clinical applications	Kang et al. (2024)
Short-term study (12 weeks)	Focus on long-term scaffold integration and degradation	Li et al. (2023a)
Short-term study	Investigate long-term integration of the bilayer scaffold	Banihashemian et al. (2024)
No long-term *in vivo* testing	Focus on *in vivo* regeneration and long-term mechanical performance	Zheng et al. (2014)
Long-term effects not assessed	Study long-term regeneration and clinical testing	Sheng et al. (2022)
Short-term study	Investigate long-term degradation and regeneration applications	Zheng et al. (2023)
Short-term study	Explore clinical translation for osteochondral defects	Wu et al. (2023)
Short-term study (12 weeks)	Optimize exosome delivery and test in larger models	Li et al. (2023b)
Short-term study; long-term effects not assessed	Explore long-term integration and optimization of stimulation	Zhang et al. (2023a)
No long-term *in vivo* testing; focused on subcutaneous models	Conduct *in vivo* testing in osteochondral defect models	You et al. (2018)
Short-term study	Investigate long-term tissue integration and scaffold degradation	Zhang et al. (2023b)
Short-term study	Investigate long-term degradation and larger animal integration	Radhakrishnan et al. (2018)
Lack of complete tissue regeneration assessment	Investigate long-term degradation and clinical translation	Zhang et al. (2021)
No *in vivo* studies; long-term effects not assessed	Focus on *in vivo* testing and scaffold optimization	Kosik-Kozioł et al. (2019)
Short-term study; no long-term assessment	Investigate long-term degradation and clinical translation	Xu et al. (2019)
No *in vivo* studies performed	Focus on *in vivo* testing and full integration for regeneration	Qin et al. (2020)
Short-term study	Focus on long-term integration and optimization for regeneration	Liu et al. (2021b)
No long-term assessment of degradation	Study long-term degradation and clinical applications	Hu et al. (2022)
Short-term study; long-term effects not assessed	Focus on long-term integration and optimization for clinical use	Li et al. (2022)
Short-term study; no long-term assessment	Investigate long-term integration and controlled release systems	Liu et al. (2020)
No *in vivo* data	Explore *in vivo* testing and clinical translation for repair	Fan et al. (2021)
Short-term study	Investigate long-term integration and clinical translation	Luo et al. (2022)
Short-term study	Focus on long-term integration and mechanical performance	Gan et al. (2019)
No *in vivo* study	Optimize scaffold for osteochondral repair with *in vivo* testing	Castro et al. (2015)
No long-term studies conducted	Focus on long-term degradation and larger model testing	Shalumon et al. (2016)
Study limited to short-term evaluation	Explore long-term integration and clinical translation	Guo et al. (2024)
Short-term animal study	Optimize hydrogel composition and test in larger models	Jin et al. (2024)

Another prevalent issue is the variability in scaffold mechanical strength, particularly when scaling up for larger defects. Achieving a mechanical resilience that closely mimics native tissue properties remains challenging. Li et al. reported that preserving compressive strength in bilayer scaffolds was difficult over long-term *in vivo* applications, highlighting a critical need for more durable biomaterials (Li et al., 2023a). Novel scaffold compositions and innovative crosslinked structures could offer the increased load-bearing capacities necessary to provide robust support in osteochondral applications, particularly those involving weight-bearing joints.

Additionally, there is limited long-term *in vivo* data on the efficacy and safety of these scaffolds. While short-term successes are frequently observed, the potential for chronic inflammation or complications related to scaffold degradation requires longer follow-up. Studies highlight the necessity for prolonged trials to thoroughly assess scaffold stability, biocompatibility, and integration with native tissue structures, all critical for achieving successful clinical translation (Brown et al., 2024; Hu et al., 2024).

To overcome these challenges, future research could focus on innovative materials and scaffold designs. The use of *in situ* forming hydrogels, which adapt to irregular defect sites during implantation, may enhance scaffold integration (Zheng et al., 2014; Park and Park, 2018; Kang et al., 2024). Smart, stimuli-responsive hydrogels capable of controlled therapeutic release could also support sustained regeneration and more effective clinical outcomes. Additionally, combining nano-hydrogels with synergistic regenerative approaches such as gene therapy, bioelectronics, or cell-based treatments may lead to multifunctional scaffolds that facilitate not only osteogenesis and chondrogenesis but also angiogenesis (Kumar et al., 2022; Chen et al., 2023). Together, these integrated approaches have the potential to advance osteochondral repair, bringing the field closer to scalable, reliable therapeutic solutions.

## 4 Conclusion

This systematic review underscores the diverse and evolving strategies employed in nano-hydrogel-based scaffolds for osteochondral repair. By systematically stratifying the included studies according to formulation type (injectable vs. preformed), structural design (single-phase, bilayered, trilayered, or gradient), and polymer origin (natural, synthetic, hybrid), we identified key trends linking scaffold architecture to biological performance. Notably, bilayered and trilayered systems that emulate the native osteochondral zonation more effectively support site-specific chondrogenesis and osteogenesis. Similarly, hybrid scaffolds integrating natural and synthetic polymers often demonstrate superior synergy between mechanical strength and bioactivity.

Despite promising preclinical outcomes, translational challenges persist. The field is hindered by variability in fabrication methods, inconsistencies in mechanical robustness and degradation profiles, and a lack of long-term *in vivo* validation. Moreover, the absence of standardized animal models and outcome measures limits direct comparison across studies, thereby impeding regulatory progression and clinical adoption.

To address these limitations, we propose a scaffold design framework emphasizing biomimetic zoning, controlled delivery of bioactive cues, stimuli-responsive behavior, and compliance with good manufacturing practice (GMP) standards. Comparative evaluations using unified scoring systems, load-bearing models, and long-term functional assessments will be critical to bridge the gap between laboratory innovation and clinical implementation.

In conclusion, while nano-hydrogels offer clear advantages in mimicking the extracellular matrix and modulating the local microenvironment, their future lies in rational design guided by translational benchmarks. With sustained interdisciplinary collaboration and regulatory foresight, these systems have the potential to evolve into clinically viable, patient-specific therapies for osteochondral regeneration.

## Data Availability

The raw data supporting the conclusions of this article will be made available by the authors, without undue reservation.

## References

[B1] Adedoyin A. Kumar R. Sridhar S. Ekenseair A. (2015). Injectable bionanocomposite hybrid scaffolds with responsive control for enhanced osteochondral tissue regeneration. IEEE, Troy, NY, USA, 1–2.

[B2] Ahmad Z. Salman S. Khan S. A. Amin A. Rahman Z. U. Al-Ghamdi Y. O. (2022). Versatility of hydrogels: from synthetic strategies, classification, and properties to biomedical applications. Gels 8 (3), 167. 10.3390/gels8030167 35323280 PMC8950628

[B3] Banihashemian A. Zamanlui Benisi S. Hosseinzadeh S. Shojaei S. Abbaszadeh H. (2024). Structural and biological investigation of alginate-nano-hydroxyapatite with chitosan-hyaluronic acid for potential osteochondral regeneration. Int. J. Polym. Mater. Polym. Biomaterials 73 (10), 851–865. 10.1080/00914037.2023.2215378

[B4] Berry D. R. Díaz B. K. Durand-Silva A. Smaldone R. A. (2019). Radical free crosslinking of direct-write 3D printed hydrogels through a base catalyzed thiol-Michael reaction. Polym. Chem. 10 (44), 5979–5984. 10.1039/c9py00953a

[B5] Brady M. A. Talvard L. Vella A. Ethier C. R. (2017). Bio‐inspired design of a magnetically active trilayered scaffold for cartilage tissue engineering. J. tissue Eng. Regen. Med. 11 (4), 1298–1302. 10.1002/term.2106 26712322

[B6] Brown N. E. Ellerbe L. R. Hollister S. J. Temenoff J. S. (2024). Development and characterization of heparin-containing Hydrogel/3D-Printed scaffold composites for craniofacial reconstruction. Ann. Biomed. Eng. 52, 2287–2307. 10.1007/s10439-024-03530-z 38734845

[B7] Cao D. Ding J. (2022). Recent advances in regenerative biomaterials. Regen. Biomater. 9, rbac098. 10.1093/rb/rbac098 36518879 PMC9745784

[B8] Cao Y. Zhang H. Qiu M. Zheng Y. Shi X. Yang J. (2024). Biomimetic injectable and bilayered hydrogel scaffold based on collagen and chondroitin sulfate for the repair of osteochondral defects. Int. J. Biol. Macromol. 257, 128593. 10.1016/j.ijbiomac.2023.128593 38056750

[B9] Cao Z. Wang H. Chen J. Zhang Y. Mo Q. Zhang P. (2023). Silk-based hydrogel incorporated with metal-organic framework nanozymes for enhanced osteochondral regeneration. Bioact. Mater. 20, 221–242. 10.1016/j.bioactmat.2022.05.025 35702612 PMC9163388

[B10] Castro N. J. Patel R. Zhang L. G. (2015). Design of a novel 3D printed bioactive nanocomposite scaffold for improved osteochondral regeneration. Cell. Mol. Bioeng. 8, 416–432. 10.1007/s12195-015-0389-4 26366231 PMC4564127

[B11] Cavendish P. A. Everhart J. S. Peters N. J. Sommerfeldt M. F. Flanigan D. C. (2019). Osteochondral allograft transplantation for knee cartilage and osteochondral defects: a review of indications, technique, rehabilitation, and outcomes. JBJS Rev. 7 (6), e7. 10.2106/jbjs.rvw.18.00123 31220000

[B12] Chahla J. Sweet M. C. Okoroha K. R. Nwachukwu B. U. Hinckel B. Farr J. (2019). Osteochondral allograft transplantation in the patellofemoral joint: a systematic review. Am. J. sports Med. 47 (12), 3009–3018. 10.1177/0363546518814236 30525887

[B13] Chander S. Kulkarni G. T. Dhiman N. Kharkwal H. (2021). Protein-based nanohydrogels for bioactive delivery. Front. Chem. 9, 573748. 10.3389/fchem.2021.573748 34307293 PMC8299995

[B14] Chen H. Huang J. Li X. Zhao W. Hua Y. Song Z. (2024). Trilayered biomimetic hydrogel scaffolds with dual-differential microenvironment for articular osteochondral defect repair. Mater. Today Bio 26, 101051. 10.1016/j.mtbio.2024.101051 PMC11021956 38633867

[B15] Chen W. Ming Y. Wang M. Huang M. Liu H. Huang Y. (2023). Nanocomposite hydrogels in regenerative medicine: applications and challenges. Macromol. Rapid Commun. 44 (15), 2300128. 10.1002/marc.202300128 37139707

[B16] Davis S. Roldo M. Blunn G. Tozzi G. Roncada T. (2021). Influence of the mechanical environment on the regeneration of osteochondral defects. Front. Bioeng. Biotechnol. 9, 603408. 10.3389/fbioe.2021.603408 33585430 PMC7873466

[B17] De Leon-Oliva D. Boaru D. L. Perez-Exposito R. E. Fraile-Martinez O. García-Montero C. Diaz R. (2023). Advanced hydrogel-based strategies for enhanced bone and cartilage regeneration: a comprehensive review. Gels 9 (11), 885. 10.3390/gels9110885 37998975 PMC10670584

[B18] Deng C. Qin C. Li Z. Lu L. Tong Y. Yuan J. (2024). Diatomite-incorporated hierarchical scaffolds for osteochondral regeneration. Bioact. Mater. 38, 305–320. 10.1016/j.bioactmat.2024.05.004 38745590 PMC11091463

[B19] Dinoro J. Maher M. Talebian S. Jafarkhani M. Mehrali M. Orive G. (2019). Sulfated polysaccharide-based scaffolds for orthopaedic tissue engineering. Biomaterials 214, 119214. 10.1016/j.biomaterials.2019.05.025 31163358

[B20] Dong Z. Yuan Q. Huang K. Xu W. Liu G. Gu Z. (2019). Gelatin methacryloyl (GelMA)-based biomaterials for bone regeneration. RSC Adv. 9 (31), 17737–17744. 10.1039/c9ra02695a 35520570 PMC9064644

[B21] Fan Z. Chen Z. Zhang H. Nie Y. Xu S. (2021). Gradient mineralized and porous double‐network hydrogel effectively induce the differentiation of BMSCs into osteochondral tissue *in vitro* for potential application in cartilage repair. Macromol. Biosci. 21 (3), 2000323. 10.1002/mabi.202000323 33356012

[B22] Filová E. Tonar Z. Lukášová V. Buzgo M. Litvinec A. Rampichová M. (2020). Hydrogel containing anti-CD44-labeled microparticles, guide bone tissue formation in osteochondral defects in rabbits. Nanomaterials 10 (8), 1504. 10.3390/nano10081504 32751860 PMC7466545

[B23] Gan D. Wang Z. Xie C. Wang X. Xing W. Ge X. (2019). Mussel‐inspired tough hydrogel with *in situ* nanohydroxyapatite mineralization for osteochondral defect repair. Adv. Healthc. Mater. 8 (22), 1901103. 10.1002/adhm.201901103 31609095

[B24] Geckil H. Xu F. Zhang X. Moon S. Demirci U. (2010). Engineering hydrogels as extracellular matrix mimics. Nanomedicine 5 (3), 469–484. 10.2217/nnm.10.12 20394538 PMC2892416

[B25] Gong L. Li J. Zhang J. Pan Z. Liu Y. Zhou F. (2020). An interleukin-4-loaded bi-layer 3D printed scaffold promotes osteochondral regeneration. Acta Biomater. 117, 246–260. 10.1016/j.actbio.2020.09.039 33007484

[B26] Gough J. E. Saiani A. Miller A. F. (2012). Peptide hydrogels: mimicking the extracellular matrix. Bioinspired, Biomim. Nanobiomaterials 1 (1), 4–12. 10.1680/bbn.11.00007

[B27] Guo C. Su Z. Zhao L. Chen R. Wang Y. Wu Y. (2024). Customized triphasic cartilage composite scaffold simulating hypoxic microenvironment for osteochondral regeneration. Compos. Part B Eng. 271, 111161. 10.1016/j.compositesb.2023.111161

[B28] Guo J. L. Kim Y. S. Koons G. L. Lam J. Navara A. M. Barrios S. (2021). Bilayered, peptide-biofunctionalized hydrogels for *in vivo* osteochondral tissue repair. Acta Biomater. 128, 120–129. 10.1016/j.actbio.2021.04.038 33930575 PMC8222183

[B29] Guo J. L. Li A. Kim Y. S. Xie V. Y. Smith B. T. Watson E. (2020). Click functionalized, tissue‐specific hydrogels for osteochondral tissue engineering. J. Biomed. Mater. Res. Part A 108 (3), 684–693. 10.1002/jbm.a.36848 PMC7942178 31755226

[B30] Hamed E. Lee Y. Jasiuk I. (2010). Multiscale modeling of elastic properties of cortical bone. Acta Mech. 213 (1), 131–154. 10.1007/s00707-010-0326-5

[B31] Hjelle K. Solheim E. Strand T. Muri R. Brittberg M. (2002). Articular cartilage defects in 1,000 knee arthroscopies. Arthrosc. J. Arthrosc. and Relat. Surg. 18 (7), 730–734. 10.1053/jars.2002.32839 12209430

[B32] Hu C. Huang R. Xia J. Hu X. Xie D. Jin Y. (2024). A nanozyme-functionalized bilayer hydrogel scaffold for modulating the inflammatory microenvironment to promote osteochondral regeneration. J. nanobiotechnology 22 (1), 445. 10.1186/s12951-024-02723-x 39069607 PMC11283693

[B33] Hu W. Wang Z. Xiao Y. Zhang S. Wang J. (2019). Advances in crosslinking strategies of biomedical hydrogels. Biomaterials Sci. 7 (3), 843–855. 10.1039/c8bm01246f 30648168

[B34] Hu X. Zheng S. Zhang R. Wang Y. Jiao Z. Li W. (2022). Dynamic process enhancement on chitosan/gelatin/nano-hydroxyapatite-bone derived multilayer scaffold for osteochondral tissue repair. Biomater. Adv. 133, 112662. 10.1016/j.msec.2022.112662 35074237

[B35] Hwang H. S. Lee C.-S. (2024). Nanoclay-composite hydrogels for bone tissue engineering. Gels 10 (8), 513. 10.3390/gels10080513 39195042 PMC11353637

[B36] Jelodari S. Ebrahimi Sadrabadi A. Zarei F. Jahangir S. Azami M. Sheykhhasan M. (2022). New insights into cartilage tissue engineering: improvement of tissue‐scaffold integration to enhance cartilage regeneration. BioMed Res. Int. 2022 (1), 7638245. 10.1155/2022/7638245 35118158 PMC8807044

[B37] Jin Y. Shu M. Liu Z. Li H. Liu C. Zhu C. (2024). Bio-functional immunomodulatory europium-doped hydroxyapatite nanorods for osteochondral repair *via* CDH5-RAS-RAF-MEK-ERK-CSF1 axis. Chem. Eng. J. 484, 149311. 10.1016/j.cej.2024.149311

[B38] Kang J. Li Y. Qin Y. Huang Z. Wu Y. Sun L. (2024). *In situ* deposition of drug and gene nanoparticles on a patterned supramolecular hydrogel to construct a directionally osteochondral plug. Nano-Micro Lett. 16 (1), 18. 10.1007/s40820-023-01228-w PMC10656386 37975889

[B39] Korpayev S. Kaygusuz G. Şen M. Orhan K. Oto Ç. Karakeçili A. (2020). Chitosan/Collagen based biomimetic osteochondral tissue constructs: a growth factor-free approach. Int. J. Biol. Macromol. 156, 681–690. 10.1016/j.ijbiomac.2020.04.109 32320808

[B40] Kosik-Kozioł A. Costantini M. Mróz A. Idaszek J. Heljak M. Jaroszewicz J. (2019). 3D bioprinted hydrogel model incorporating β-tricalcium phosphate for calcified cartilage tissue engineering. Biofabrication 11 (3), 035016. 10.1088/1758-5090/ab15cb 30943457

[B41] Kumar A. Sood A. Singhmar R. Mishra Y. K. Thakur V. K. Han S. S. (2022). Manufacturing functional hydrogels for inducing angiogenic–osteogenic coupled progressions in hard tissue repairs: prospects and challenges. Biomaterials Sci. 10 (19), 5472–5497. 10.1039/d2bm00894g 35994005

[B42] Lan W. Xu M. Qin M. Cheng Y. Zhao Y. Huang D. (2021). Physicochemical properties and biocompatibility of the bi-layer polyvinyl alcohol-based hydrogel for osteochondral tissue engineering. Mater. and Des. 204, 109652. 10.1016/j.matdes.2021.109652

[B43] Lee J. H. (2018). Injectable hydrogels delivering therapeutic agents for disease treatment and tissue engineering. Biomaterials Res. 22 (1), 27. 10.1186/s40824-018-0138-6 PMC6158836 30275970

[B44] Li C. Zhang W. Nie Y. Jiang D. Jia J. Zhang W. (2023a). Integrated and bifunctional bilayer 3D printing scaffold for osteochondral defect repair. Adv. Funct. Mater. 33 (20), 2214158. 10.1002/adfm.202214158

[B45] Li M. Song P. Wang W. Xu Y. Li J. Wu L. (2022). Preparation and characterization of biomimetic gradient multi-layer cell-laden scaffolds for osteochondral integrated repair. J. Mater. Chem. B 10 (22), 4172–4188. 10.1039/d2tb00576j 35531933

[B46] Li Q. Yu H. Zhao F. Cao C. Wu T. Fan Y. (2023b). 3D printing of microenvironment‐specific bioinspired and exosome‐reinforced hydrogel scaffolds for efficient cartilage and subchondral bone regeneration. Adv. Sci. 10 (26), 2303650. 10.1002/advs.202303650 PMC10502685 37424038

[B47] Liu K. Liu Y. Duan Z. Ma X. Fan D. (2021a). A biomimetic bi-layered tissue engineering scaffolds for osteochondral defects repair. Sci. China Technol. Sci. 64 (4), 793–805. 10.1007/s11431-020-1597-4

[B48] Liu P. Li M. Yu H. Fang H. Yin J. Zhu D. (2021b). Biphasic CK2. 1-coated β-glycerophosphate chitosan/LL37-modified layered double hydroxide chitosan composite scaffolds enhance coordinated hyaline cartilage and subchondral bone regeneration. Chem. Eng. J. 418, 129531. 10.1016/j.cej.2021.129531

[B49] Liu X. Chen Y. Mao A. S. Xuan C. Wang Z. Gao H. (2020). Molecular recognition-directed site-specific release of stem cell differentiation inducers for enhanced joint repair. Biomaterials 232, 119644. 10.1016/j.biomaterials.2019.119644 31884017

[B50] Liu Y. Hsu S.-H. (2018). Synthesis and biomedical applications of self-healing hydrogels. Front. Chem. 6, 449. 10.3389/fchem.2018.00449 30333970 PMC6176467

[B51] Lowen J. M. Wheeler E. E. Shimamoto N. K. Ramos‐Rodriguez D. H. Griffin K. H. Bond G. C. (2024). Functionalized annealed microgels for spatial control of osteogenic and chondrogenic differentiation. Adv. Funct. Mater. 34, 2311017. 10.1002/adfm.202311017 PMC12341486 40800235

[B52] Luo M. Chen M. Bai J. Chen T. He S. Peng W. (2022). A bionic composite hydrogel with dual regulatory functions for the osteochondral repair. Colloids Surfaces B Biointerfaces 219, 112821. 10.1016/j.colsurfb.2022.112821 36108368

[B53] Lynch C. R. Kondiah P. P. Choonara Y. E. (2021). Advanced strategies for tissue engineering in regenerative medicine: a biofabrication and biopolymer perspective. Molecules 26 (9), 2518. 10.3390/molecules26092518 33925886 PMC8123515

[B54] Macleod M. R. O’collins T. Howells D. W. Donnan G. A. (2004). Pooling of animal experimental data reveals influence of study design and publication bias. Stroke 35 (5), 1203–1208. 10.1161/01.str.0000125719.25853.20 15060322

[B55] Mano J. Reis R. (2007). Osteochondral defects: present situation and tissue engineering approaches. J. tissue Eng. Regen. Med. 1 (4), 261–273. 10.1002/term.37 18038416

[B56] Martin I. Miot S. Barbero A. Jakob M. Wendt D. (2007). Osteochondral tissue engineering. J. biomechanics 40 (4), 750–765. 10.1016/j.jbiomech.2006.03.008 16730354

[B57] Moher D. Liberati A. Tetzlaff J. Altman D. G. Prisma G. T. (2009). Preferred reporting items for systematic reviews and meta-analyses: the PRISMA statement. Ann. Intern. Med. 151 (4), 264–269. 10.7326/0003-4819-151-4-200908180-00135 19622511

[B58] Moher D. Shamseer L. Clarke M. Ghersi D. Liberati A. Petticrew M. (2015). Preferred reporting items for systematic review and meta-analysis protocols (PRISMA-P) 2015 statement. Syst. Rev. 4, 1–9. 10.1186/2046-4053-4-1 25554246 PMC4320440

[B59] Nguyen L. H. Kudva A. K. Saxena N. S. Roy K. (2011). Engineering articular cartilage with spatially-varying matrix composition and mechanical properties from a single stem cell population using a multi-layered hydrogel. Biomaterials 32 (29), 6946–6952. 10.1016/j.biomaterials.2011.06.014 21723599

[B60] Park K. M. Park K. D. (2018). *In situ* cross-linkable hydrogels as a dynamic matrix for tissue regenerative medicine. Tissue Eng. Regen. Med. 15, 547–557. 10.1007/s13770-018-0155-5 30603578 PMC6171695

[B61] Paul A. Manoharan V. Krafft D. Assmann A. Uquillas J. A. Shin S. R. (2016). Nanoengineered biomimetic hydrogels for guiding human stem cell osteogenesis in three dimensional microenvironments. J. Mater. Chem. B 4 (20), 3544–3554. 10.1039/c5tb02745d 27525102 PMC4980085

[B62] Qiao Z. Lian M. Han Y. Sun B. Zhang X. Jiang W. (2021). Bioinspired stratified electrowritten fiber-reinforced hydrogel constructs with layer-specific induction capacity for functional osteochondral regeneration. Biomaterials 266, 120385. 10.1016/j.biomaterials.2020.120385 33120203

[B63] Qin Y. Li G. Wang C. Zhang D. Zhang L. Fang H. (2020). Biomimetic bilayer scaffold as an incubator to induce sequential chondrogenesis and osteogenesis of adipose derived stem cells for construction of osteochondral tissue. ACS Biomaterials Sci. and Eng. 6 (5), 3070–3080. 10.1021/acsbiomaterials.0c00200 33463252

[B64] Quazi M. Z. Park N. (2022). Nanohydrogels: advanced polymeric nanomaterials in the era of nanotechnology for robust functionalization and cumulative applications. Int. J. Mol. Sci. 23 (4), 1943. 10.3390/ijms23041943 35216058 PMC8875080

[B65] Radhakrishnan J. Manigandan A. Chinnaswamy P. Subramanian A. Sethuraman S. (2018). Gradient nano-engineered *in situ* forming composite hydrogel for osteochondral regeneration. Biomaterials 162, 82–98. 10.1016/j.biomaterials.2018.01.056 29438883

[B66] Rana M. M. De La Hoz Siegler H. (2024). Evolution of hybrid hydrogels: next-generation biomaterials for drug delivery and tissue engineering. Gels 10 (4), 216. 10.3390/gels10040216 38667635 PMC11049329

[B67] Sethi S. Medha T. S. Singh A. Kaith B. S. Khullar S. (2023). Handbook of green and sustainable nanotechnology: fundamentals, developments and applications. Springer Nature, 1–31.

[B68] Shalumon K. Sheu C. Fong Y. T. Liao H.-T. Chen J.-P. (2016). Microsphere-based hierarchically juxtapositioned biphasic scaffolds prepared from poly (lactic-co-glycolic acid) and nanohydroxyapatite for osteochondral tissue engineering. Polymers 8 (12), 429. 10.3390/polym8120429 30974705 PMC6431887

[B69] Sheng R. Chen J. Wang H. Luo Y. Liu J. Chen Z. (2022). Nanosilicate‐reinforced silk fibroin hydrogel for endogenous regeneration of both cartilage and subchondral bone. Adv. Healthc. Mater. 11 (17), 2200602. 10.1002/adhm.202200602 35749970

[B70] Soni S. S. D'elia A. M. Alsasa A. Cho S. Tylek T. O'brien E. M. (2022). Sustained release of drug-loaded nanoparticles from injectable hydrogels enables long-term control of macrophage phenotype. Biomaterials Sci. 10 (24), 6951–6967. 10.1039/d2bm01113a PMC9724601 36341688

[B71] Suvarnapathaki S. Wu X. Lantigua D. Nguyen M. A. Camci‐Unal G. (2020). Hydroxyapatite‐incorporated composite gels improve mechanical properties and bioactivity of bone scaffolds. Macromol. Biosci. 20 (10), 2000176. 10.1002/mabi.202000176 32755044

[B72] Tomal W. Ortyl J. (2020). Water-soluble photoinitiators in biomedical applications. Polymers 12 (5), 1073. 10.3390/polym12051073 32392892 PMC7285382

[B73] Tortorici M. Petersen A. Duda G. N. Checa S. (2022). The degradation of synthetic polymeric scaffolds with strut-like architecture influences the mechanics-dependent repair process of an osteochondral defect *in silico* . Front. Bioeng. Biotechnol. 10, 846665. 10.3389/fbioe.2022.846665 35360392 PMC8960607

[B74] Verhagen R. A. Struijs P. A. Bossuyt P. M. Van Dijk C. N. (2003). Systematic review of treatment strategies for osteochondral defects of the talar dome. Foot ankle Clin. 8 (2), 233–242. 10.1016/s1083-7515(02)00064-5 12911238

[B75] Vilela C. Correia C. Oliveira J. M. Sousa R. A. Espregueira-Mendes J. Reis R. L. (2015). Cartilage repair using hydrogels: a critical review of *in vivo* experimental designs. ACS Biomaterials Sci. and Eng. 1 (9), 726–739. 10.1021/acsbiomaterials.5b00245 33445249

[B76] Wang H. Hu B. Li H. Feng G. Pan S. Chen Z. (2022a). Biomimetic mineralized hydroxyapatite nanofiber-incorporated methacrylated gelatin hydrogel with improved mechanical and osteoinductive performances for bone regeneration. Int. J. Nanomedicine Vol. 17, 1511–1529. 10.2147/ijn.s354127 PMC8978691 35388269

[B77] Wang S. Qiu Y. Qu L. Wang Q. Zhou Q. (2022b). Hydrogels for treatment of different degrees of osteoarthritis. Front. Bioeng. Biotechnol. 10, 858656. 10.3389/fbioe.2022.858656 35733529 PMC9207401

[B78] Wei G. Ma P. X. (2008). Nanostructured biomaterials for regeneration. Adv. Funct. Mater. 18 (22), 3568–3582. 10.1002/adfm.200800662 PMC2701700 19946357

[B79] Wu H. Shang Y. Sun W. Ouyang X. Zhou W. Lu J. (2023). Seamless and early gap healing of osteochondral defects by autologous mosaicplasty combined with bioactive supramolecular nanofiber-enabled gelatin methacryloyl (BSN-GelMA) hydrogel. Bioact. Mater. 19, 88–102. 10.1016/j.bioactmat.2022.03.038 35441114 PMC9005961

[B80] Xiang C. Zhang X. Zhang J. Chen W. Li X. Wei X. (2022). A porous hydrogel with high mechanical strength and biocompatibility for bone tissue engineering. J. Funct. Biomaterials 13 (3), 140. 10.3390/jfb13030140 PMC9504119 36135575

[B81] Xu J. Feng Q. Lin S. Yuan W. Li R. Li J. (2019). Injectable stem cell-laden supramolecular hydrogels enhance *in situ* osteochondral regeneration via the sustained co-delivery of hydrophilic and hydrophobic chondrogenic molecules. Biomaterials 210, 51–61. 10.1016/j.biomaterials.2019.04.031 31075723

[B82] Yan L.-P. Silva-Correia J. Oliveira M. B. Vilela C. Pereira H. Sousa R. A. (2015). Bilayered Silk/silk-nanoCaP scaffolds for osteochondral tissue engineering: *in vitro* and *in vivo* assessment of biological performance. Acta Biomater. 12, 227–241. 10.1016/j.actbio.2014.10.021 25449920

[B83] Yang W. Zhu P. Huang H. Zheng Y. Liu J. Feng L. (2019). Functionalization of novel theranostic hydrogels with kartogenin-grafted USPIO nanoparticles to enhance cartilage regeneration. ACS Appl. Mater. and interfaces 11 (38), 34744–34754. 10.1021/acsami.9b12288 31475824

[B84] Yang Y. Xu T. Zhang Q. Piao Y. Bei H. P. Zhao X. (2021). Biomimetic, stiff, and adhesive periosteum with osteogenic–angiogenic coupling effect for bone regeneration. Small 17 (14), 2006598. 10.1002/smll.202006598 33705605

[B85] Yao H. Wang C. Zhang Y. Wan Y. Min Q. (2023). Manufacture of bilayered composite hydrogels with strong, elastic, and tough properties for osteochondral repair applications. Biomimetics 8 (2), 203. 10.3390/biomimetics8020203 37218789 PMC10204375

[B86] You B. Li Q. Dong H. Huang T. Cao X. Liao H. (2018). Bilayered HA/CS/PEGDA hydrogel with good biocompatibility and self-healing property for potential application in osteochondral defect repair. J. Mater. Sci. and Technol. 34 (6), 1016–1025. 10.1016/j.jmst.2017.11.016

[B87] Yue S. He H. Li B. Hou T. (2020). Hydrogel as a biomaterial for bone tissue engineering: a review. Nanomaterials 10 (8), 1511. 10.3390/nano10081511 32752105 PMC7466535

[B88] Zengin A. Castro J. Habibovic P. Van Rijt S. (2021). Injectable, self-healing mesoporous silica nanocomposite hydrogels with improved mechanical properties. Nanoscale 13 (2), 1144–1154. 10.1039/d0nr07406c 33400753 PMC8100892

[B89] Zhang H. Huang H. Hao G. Zhang Y. Ding H. Fan Z. (2021). 3D printing hydrogel scaffolds with nanohydroxyapatite gradient to effectively repair osteochondral defects in rats. Adv. Funct. Mater. 31 (1), 2006697. 10.1002/adfm.202006697

[B90] Zhang H. Li Q. Xu X. Zhang S. Chen Y. Yuan T. (2022a). Functionalized microscaffold–hydrogel composites accelerating osteochondral repair through endochondral ossification. ACS Appl. Mater. and interfaces 14 (47), 52599–52617. 10.1021/acsami.2c12694 36394998

[B91] Zhang L. Dai W. Gao C. Wei W. Huang R. Zhang X. (2023a). Multileveled hierarchical hydrogel with continuous biophysical and biochemical gradients for enhanced repair of full‐thickness osteochondral defect. Adv. Mater. 35 (19), 2209565. 10.1002/adma.202209565 36870325

[B92] Zhang P. Chen J. Sun Y. Cao Z. Zhang Y. Mo Q. (2023b). A 3D multifunctional bi-layer scaffold to regulate stem cell behaviors and promote osteochondral regeneration. J. Mater. Chem. B 11 (6), 1240–1261. 10.1039/d2tb02203f 36648128

[B93] Zhang Y. Dong Q. Zhao X. Sun Y. Lin X. Zhang X. (2024). Honeycomb-like biomimetic scaffold by functionalized antibacterial hydrogel and biodegradable porous Mg alloy for osteochondral regeneration. Front. Bioeng. Biotechnol. 12, 1417742. 10.3389/fbioe.2024.1417742 39070169 PMC11273084

[B94] Zhang Y. Han Y. Peng Y. Lei J. Chang F. (2022b). Bionic biphasic composite scaffolds with osteochondrogenic factors for regeneration of full-thickness osteochondral defects. Biomaterials Sci. 10 (7), 1713–1723. 10.1039/d2bm00103a 35229096

[B95] Zheng L. Jiang X. Chen X. Fan H. Zhang X. (2014). Evaluation of novel *in situ* synthesized nano-hydroxyapatite/collagen/alginate hydrogels for osteochondral tissue engineering. Biomed. Mater. 9 (6), 065004. 10.1088/1748-6041/9/6/065004 25358331

[B96] Zheng S. Li D. Liu Q. Tang C. Hu W. Ma S. (2023). Surface-modified nano-hydroxyapatite uniformly dispersed on high-porous GelMA scaffold surfaces for enhanced osteochondral regeneration. Int. J. Nanomedicine Vol. 18, 5907–5923. 10.2147/ijn.s428965 PMC10599329 37886722

[B97] Zhu D. Tong X. Trinh P. Yang F. (2018). Mimicking cartilage tissue zonal organization by engineering tissue-scale gradient hydrogels as 3D cell niche. Tissue Eng. Part A 24 (1-2), 1–10. 10.1089/ten.tea.2016.0453 28385124 PMC5770099

