# Supplementary material for: The current status of nano-hydrogel preparations for osteochondral repair: Systematic Review
Source: Front Bioeng Biotechnol. 2025 Jul 1;13:1611522. doi: 10.3389/fbioe.2025.1611522 (PMC12259566; doi:10.3389/fbioe.2025.1611522)
Supplement: Supplementary file 1 [file Table1.docx]

# SUPPORTING INFORMATION

**Table 1s. Database-Specific Search Strategies**
Detailed search terms and strategies applied to each database using relevant keywords.

| **PUBMED search** | ((((((((((((((Nanohydrogel[MeSH Terms]) OR (nanogel[MeSH Terms])) OR (nano-hydrogel scaffold[MeSH Terms])) OR (nanoscale Hydrogels [MeSH Terms])) OR (nano-sized Hydrogels [MeSH Terms])) OR (nano composite hydrogel[MeSH Terms])) OR (nano structured hydrogel[MeSH Terms])) OR (nanogel scaffold[MeSH Terms])) OR (nano fibrous hydrogel[MeSH Terms])) OR (nanoparticle-loaded hydrogel [MeSH Terms])) OR (Nanoparticulate Hydrogels[MeSH Terms])) OR (Submicron Hydrogels[MeSH Terms]))AND (osteochondral repair[MeSH Terms])) OR (cartilage restoration[MeSH Terms])) OR (cartilage regeneration [MeSH Terms])) OR (cartilage repair [MeSH Terms])) OR (cartilage tissue engineering [MeSH Terms])) OR (Cartilage and Bone Restoration [MeSH Terms])) OR (Joint Repair [MeSH Terms])) OR (Osteochondral Restoration [MeSH Terms])) OR (Chondral and Osseous Repair [MeSH Terms])) OR (Articular Surface Repair [MeSH Terms])) OR (Osteochondral Regeneration [MeSH Terms])) OR (Joint Tissue Reconstruction [MeSH Terms])) OR (Cartilage and Bone Healing [MeSH Terms])) |
| --- | --- |
| **Scopus** | TITLE-ABS-KEY ("nanohydrogel" OR "nanogel" OR "nano-hydrogel scaffold" OR "Nanoscale Hydrogels" OR "Nano-sized Hydrogels" OR "Nanocomposite Hydrogels" OR "Nanostructured Hydrogels" OR "Nanogel Scaffolds" OR "Nanofibrous Hydrogels" OR "Nanoparticle-loaded Hydrogels" OR "Nanoparticulate Hydrogels" OR "Submicron Hydrogels") AND ( "osteochondral repair" OR "cartilage restoration" OR "cartilage regeneration" OR "cartilage repair" OR "cartilage tissue engineering" OR "Cartilage and Bone Restoration" OR "Joint Repair" OR "Osteochondral Restoration" OR "Chondral and Osseous Repair" OR "Articular Surface Repair" OR "Osteochondral Regeneration" OR "Joint Tissue Reconstruction" OR "Cartilage and Bone Healing" ) AND PUBYEAR > 2011 AND PUBYEAR < 2025 AND ( LIMIT-TO ( SRCTYPE , "j" ) ) AND ( LIMIT-TO ( DOCTYPE , "ar" ) ) AND ( LIMIT-TO ( LANGUAGE , "English" ) ) |
| **Web of Science** | (TS = Nanohydrogel OR TS = nanogel OR TS = nano-hydrogel scaffold OR TS =Nanoscale Hydrogels OR TS = Nano-sized Hydrogels OR TS = nano composite hydrogel OR TS = nano structured hydrogel OR TS = nanogel scaffold OR TS = nano fibrous hydrogel OR TS = nanoparticle-loaded hydrogel OR TS = Nanoparticulate Hydrogels OR TS = Submicron Hydrogels) AND (TS = osteochondral repair OR TS = cartilage restoration OR TS = cartilage regeneration OR TS = cartilage repair OR TS = cartilage tissue engineering OR TS = Cartilage and Bone Restoration OR TS = Joint Repair OR TS = Osteochondral Restoration OR TS = Chondral and Osseous Repair OR TS = Articular Surface Repair OR TS = Osteochondral Regeneration OR TS = Joint Tissue Reconstruction OR TS = Cartilage and Bone Healing), English |
